# Activating PKC-ε induces HIV expression with improved tolerability

**DOI:** 10.1371/journal.ppat.1012874

**Published:** 2025-02-06

**Authors:** Alivelu M. Irrinki, Jasmine Kaur, Bally Randhawa, Ryan McFadden, Chelsea Snyder, Hoa Truong, Daniel Soohoo, Eric Hu, Helen Yu, Bernard P. Murray, Bing Lu, Dmytro Kornyeyev, Ishak Darryl Irwan, Lan Nguyen, Yu-San Yang, Jean-Philippe Belzile, Uli Schmitz, Todd C. Appleby, Brian Schultz, Jay Lalezari, Steven Deeks, Tomas Cihlar, Jeffrey P. Murry

**Affiliations:** 1 Gilead Sciences, Inc., Foster City, California, United States of America; 2 Quest Clinical Research, San Francisco, California, United States of America; 3 Department of Medicine, University of California, San Francisco, San Francisco, California, United States of America; Johns Hopkins University School of Medicine, UNITED STATES OF AMERICA

## Abstract

Despite suppressive antiretroviral therapy (ART), HIV-1 persists in latent reservoirs that seed new HIV infections if ART is interrupted, necessitating lifelong therapy for people with HIV. Activation of latent HIV during ART could improve recognition and elimination of infected cells by the immune system. Protein kinase C (PKC) isozymes increase HIV transcription and hence are potential latency reversal agents. However, the clinical utility of PKCs for this application is limited due to toxicity, which is poorly understood. Our studies showed that PKC activation with multiple classes of agonists leads to widespread platelet activation, consistent with disseminated intravascular coagulation, at concentrations that were similar to those required for T-cell activation. Differential expression analysis indicated that PKC-ε and PKC-η isoforms are expressed at high levels in human CD4^+^ T cells but not in platelets. Using structure-based drug design, we developed a novel PKC agonist, C-233, with increased selectivity for PKC-ε. C-233 increased both supernatant HIV RNA and p24 expression ex vivo after treatment of CD4^+^ T cells from ART-suppressed people with HIV. C-233 was 5-fold more potent for T-cell activation relative to platelet activation. Our studies support the use of structure-based drug design to create selective novel PKC agonists for the safe activation of HIV reservoirs and improved tolerability.

## Author summary

Elimination of the HIV-1 reservoir is a key step in a therapeutic strategy leading to HIV drug-free remission and/or cure. Activation of latent HIV could improve recognition and eventual clearance of persistent HIV by the immune system. Protein kinase C (PKC) agonists are highly effective at activating latent HIV expression but are limited by safety concerns and an insufficient understanding of the mechanism of their adverse effects. In this study, we show that the currently available PKC agonists cause widespread platelet activation, leading to a severe syndrome that closely resembles disseminated intravascular coagulation. However, agonists with increased PKC-ε selectivity had improved in vivo tolerability relative to the other agonists. Using structure-based drug design, we developed a molecule, C-233, which has increased selectivity for PKC-ε, reduced platelet toxicity, and strongly activates latent HIV RNA and viral protein expression. This novel strategy for improving PKC agonist safety may help advance this class of compounds as part of a therapeutic strategy leading to HIV drug-free remission and/or cure.

## Introduction

Latent HIV reservoirs represent the major barrier to an effective cure for HIV infection [1]. Contemporary antiretroviral therapy (ART) targets multiple steps in the HIV life cycle, including viral entry, integration, replication, and assembly, but does not eradicate proviral reservoirs. These reservoirs typically initiate viral rebound if ART is interrupted. As a result, people living with HIV must maintain lifelong treatment to suppress viral replication, which is associated with challenges related to long-term access and adherence, tolerability, chronic inflammation, and drug–drug interactions. The development of a safe, effective curative intervention remains a global priority [2].

Efforts to target latent reservoirs have been hampered by low antigen expression, which makes latently infected cells refractory to immune-mediated clearance [3,4]. Although several strategies for activating viral expression have been proposed, the clinical utility of the latency reversal agents (LRAs) assessed to date has been limited by toxicity [5]. LRAs that have advanced to clinical trials have shown low-level increases in viral transcription without significant viral reservoir perturbation [6–8]. Thus, there is a need to identify potent and safe LRAs that can facilitate the efficient elimination of HIV-1 reservoirs [5].

Protein kinase C (PKC) isozymes belonging to a family of phospholipid-dependent serine-threonine kinases are actively engaged in several signal transduction pathways and physiological cellular responses through phosphorylation of multiple proteins [9]. They act as therapeutic targets for diseases including cancers and disorders of cardiovascular, inflammatory, neurological, and metabolic systems [10–17]. PKC isozymes have been classified into 3 subtypes based on protein subdomain arrangement: classical PKCs (cPKCs: α, β, and γ), novel PKCs (nPKCs: δ, ε, η, and θ), and atypical PKCs (aPKCs: ι and ζ). Both cPKCs and nPKCs bind 1,2-diacylglycerol (DAG), an endogenous second messenger downstream of phospholipase C. DAG binding induces PKC activation, membrane translocation, and target protein phosphorylation [18].

Based on their critical role in cell signaling, PKC agonists are being evaluated preclinically and clinically for treatment of neurologic disorders such as Alzheimer’s disease, various cancers, and HIV infection [11–13,16,17,19]. PKC agonists activate multiple transcription factors that increase HIV transcription, such as NF-κB, NF-AT, and AP-1 [20–23]. As a result, PKC agonists are among the most effective LRAs described to date [24–26]. Most PKC agonists that have been studied are complex natural products that are difficult to synthesize chemically [24]. By contrast, diacylglycerol lactones (DAG-lactones) are synthetically tractable PKC agonists that act as mimics of DAG, the endogenous ligand for cPKCs and nPKCs [27]. Several DAG-lactones can activate HIV expression and synergize with other classes of LRAs in vitro [28].

Multiple PKC agonists have been evaluated both in vitro and in vivo; however, in vivo studies have been limited by safety concerns. Many studies have shown that compounds such as bryostatin, ingenol mebutate, and prostratin strongly activate in vitro and ex vivo HIV expression, but very few reports have addressed their in vivo toxicity profile [29–34]. In a clinical trial, a well-tolerated low dose of bryostatin-1 had no measurable effect on PKC activity or HIV transcription, but exposures achieved were well below those reported to be required for HIV activation [17]. Clinical approval of ingenol mebutate was initially restricted to topical application in order to avoid systemic toxicity, but approval was withdrawn over safety concerns [35]. Systemic dosing of prostratin induced dose-dependent toxicity and mortality at levels required to induce immune activation in rodent models [36]. These results indicate that novel strategies for improving PKC agonist safety are required to advance this class of compounds for HIV therapies.

Despite the large body of work showing that PKC agonists activate HIV transcription, much less is known about the mechanisms of PKC agonist toxicity in vivo. In this study, we show that PKC activation in vivo leads to widespread platelet activation, consistent with disseminated intravascular coagulation (DIC). This activity is broadly associated with multiple classes of nonselective PKC agonists and is consistent with high expression of multiple PKC isoforms in platelets. We hypothesized that PKC agonist toxicity could be improved by selectively activating PKC isoforms that have low platelet expression. We show that PKC-ε and PKC-η have low expression in platelets and strong expression in T cells. We designed and synthesized a novel PKC agonist that has increased selectivity for PKC-ε. This agonist activated T cells in vivo at doses that had little effect on platelets. This work supports continued structure-based design of selective PKC agonists for safe activation of HIV expression.

## Results

### Novel PKC isoforms are sufficient for activation of HIV expression

**Fig 1:**
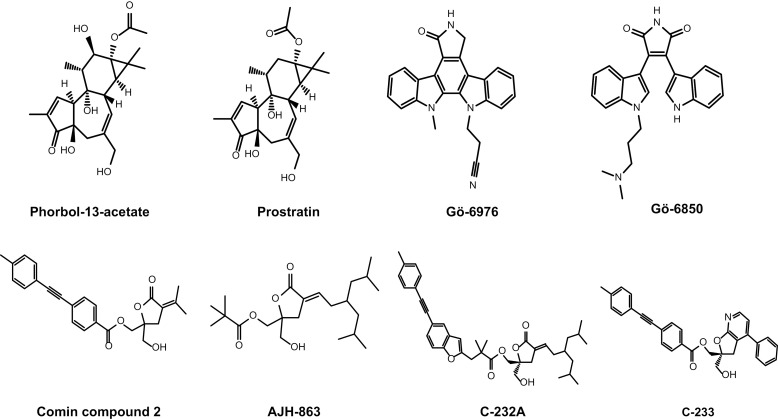
PKC agonists and other compounds used in this study.

**Fig 2:**
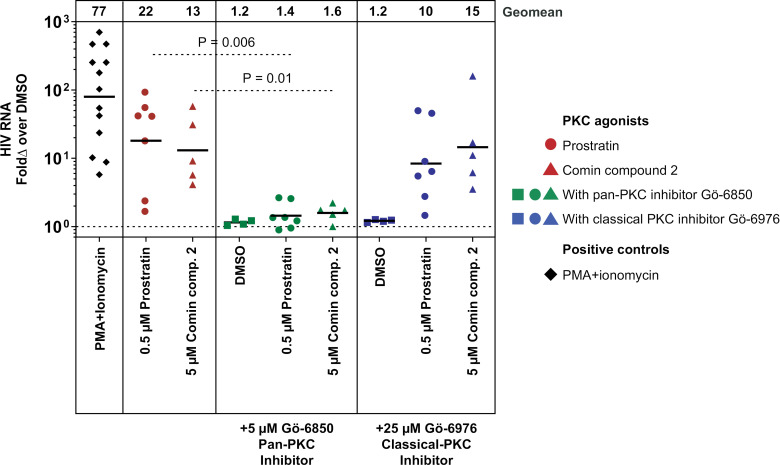
HIV activation by novel PKC isoforms. HIV viral RNA (copies/mL) fold change over DMSO (vehicle). Total CD4^+^ T cells from ART-suppressed people with HIV (n = 12) treated with PKC agonists [prostratin (red circles, n = 7)/Comin compound 2 (red triangles, n = 5)] alone, with pan-PKC inhibitor Gӧ-6850 (green circles/green triangles), or with classical PKC inhibitor Gӧ-6976 (blue circles/blue triangles). PMA and ionomycin (black diamonds) were used as positive control. Each symbol represents the geometric mean for a single individual (n = 3 to 8 replicate wells). Horizontal bars indicate the geometric means of all individuals in the indicated condition. Horizontal line on y-axis indicates the normalized value for DMSO. Significant differences were determined by ratio-paired *t* test.

Most PKC agonists broadly activate classical and novel PKC isoforms. To determine which of these 2 classes of PKC isoforms is sufficient for activation of HIV expression by PKC agonists, we treated total CD4^+^ T cells isolated from ART-suppressed people with HIV with PKC agonists, in the presence or absence of pan-PKC and classical-PKC inhibitors, Gӧ-6850 and Gӧ-6976, respectively for 4 days (Figs 1, 2, and S1). We initially tested 2 PKC agonists: prostratin (n = 7) and a DAG-lactone designed by Comin and colleagues (designated here as Comin compound 2) (n = 5) [37]. Both PKC agonists robustly induced supernatant HIV. While treatment with the pan PKC inhibitor Gӧ-6850 abrogated HIV activation, treatment with the classical PKC inhibitor, Gӧ-6976, did not significantly change the effect of prostratin or Comin compound 2. These data indicate that novel PKC isoforms are sufficient for HIV activation.

A biochemical Z’-LYTE kinase activity assay (Thermo Fisher Scientific) was utilized to verify the specificity of Gӧ-6850 and Gӧ-6976 (Table 1). In this assay, phosphorylation of a synthetic FRET-peptide substrate protects the peptide from cleavage by the development reagent and prevents disruption of the FRET signal. Gӧ-6850 and Gӧ-6976 were tested in a 10-point dose response with 3-fold serial dilutions and half-maximal inhibitory concentration (IC_50_) values were determined. Based on these results, we estimated that 5 µM Gӧ-6850 (46% free in cell culture media) would inhibit >90% of all classical and novel isoforms, except for PKC-ε, which would be 70% inhibited. We also estimated that 25 µM Gӧ-6976 (34% free in cell culture media) would inhibit 75%–95% of classical isoform activity without impacting novel PKC isoforms.

**Table 1:** Selectivity of PKC inhibitors towards PKC isoforms^a^.

	IC_50_ for classical PKC isoforms (nM)				IC_50_ for novel PKC isoforms (nM)			
**Inhibitor**	**PKC-α**	**PKC-β1**	**PKC-β2**	**PKC-γ**	**PKC-δ**	**PKC-ε**	**PKC-η**	**PKC-θ**
Gӧ-6850 (pan)	2.53	0.457	0.331	2.18	9.63	67.6	11.3	3
Gӧ-6976 (classical)	101	469	182	136	15,000	15,000	15,000	15,000

^a^IC_50_ values were determined using ATP concentration at the Kmapp for each enzyme and 2 μM peptide substrates.

We evaluated the sufficiency of individual novel PKC isoforms for activating HIV expression in a Jurkat reporter cell line containing latent HIV and constitutively active PKC-ε, -η, -θ, or -δ isoforms [38–42]. HIV activation was detected after transfection with plasmids encoding PKC-ε, -η, or -θ as well as the treatment with 0.4 µM prostratin as a positive control (S2A and S2B Fig). In a different study, we used HEK293 cell lines containing NF-kB or AP1-inducible luciferase reporters that were activated by individual novel PKC isoforms. The strongest responses were observed after transfection of PKC-ε and -θ (S2C and S2D Fig). This is in line with conclusions made based on the results for selective inhibitors and that novel PKC isoforms alone can lead to activation of latent HIV.

### The PKC agonist C-232A activates novel PKC isoforms and HIV expression

We designed a DAG-lactone with selectivity for novel PKC isoforms to further test whether these isoforms are sufficient for latency reversal. Our efforts toward more selective PKC agonists extended the work by Marquez et al. [43–45] and the liganded PKC crystal structure of phorbol-13-acetate bound to the C1B domain of PKC-δ (PDB id 1PTR) [46]. Marquez et al. established that constrained versions of DAG-lactones resulting from modification of the ester moiety (as seen in Comin compound 2) [37] and alkyl groups (as seen in AJH-863) maintain most of the polar interactions seen in the phorbol-PKC-δ C1B structure [27]. AJH-863, in particular, emerged as the most potent PKC agonist with low nanomolar potency and an approximately 10-fold selectivity for δ and ε over α and β (Fig 1) [47]. We sought to determine the crystal structure of AJH-863 bound to PKC-δ in order to gain a more detailed understanding beyond the general effects of increasing alkyl substituents, especially of how its branched dimethyl-heptane grouping leads to enhanced potency and selectivity.

**Fig 3:**
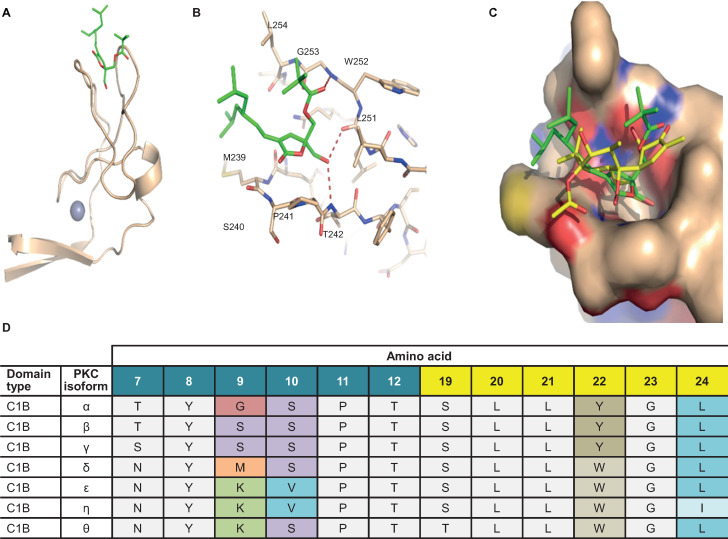
Crystal structure of DAG-lactone bound to PKC-δ. (A) Structure of the novel small-molecule PKC agonist, AJH-863 (stick model with green carbon atoms), bound to the PKC-δ C1B domain (ribbon model with zinc atom [purple]). (B) Detailed interactions of AJH-863 (stick model with green carbon atoms) with the C1B domain. Specific hydrogen bonds are displayed as red dashed lines. (C) Overlay of AJH-863 (stick model with green carbon atoms) and phorbol-13-acetate (stick model with yellow carbon atoms) in the C1B binding site. The protein is displayed as a surface and colored by atom type with beige carbons. (D) Sequence alignment of select C1B residues across PKC isoforms.

The crystal structure confirmed that the hydroxymethyl group binds deep into a groove formed by 2 loops consisting of residues 239–242 and residues 251–254, respectively (Fig 3A and 3B). The hydroxyl group donates a hydrogen bond to the backbone carbonyl oxygen of Leu251 and accepts a hydrogen bond from the backbone amide nitrogen of Thr242. The lactone ring packs against the side chain of Pro241, while the branched lipophilic group packs against residues Met239 and Leu254. The carbonyl oxygen of the ester linkage forms a slightly longer hydrogen bond (3.2 Å) to the backbone amide nitrogen of Gly253. The tert-butyl capping group extends away from the binding pocket of C1B toward the solvent in the crystal structure. In conclusion, AJH-863 binds to the C1B domain of PKC-δ in a similar fashion as phorbol-13-acetate (PDB id:1PTR) (Fig 3C) [46].

**Fig 4:**
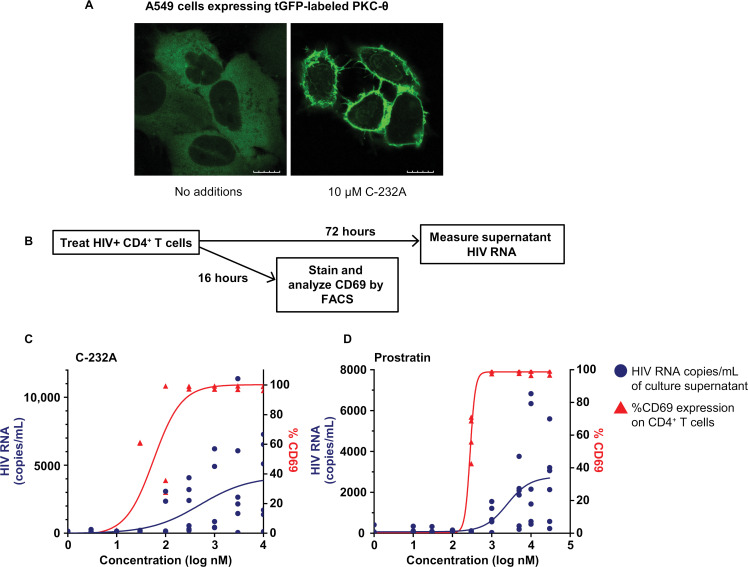
PKC-θ and HIV expression activation by novel PKC agonist, C-232A. (A) Confocal microscopy images of A549 cells expressing tGFP-labeled PKC-θ isoform showing translocation of PKC-θ from cytoplasm to cell edge upon treatment with C-232A. Scale bar represents 10 µm. (B) Assay workflow to measure CD69 and HIV activation in CD4^+^ T cells isolated from ART-suppressed people with HIV. CD69 expression on CD4^+^ T cells (n = 4) and HIV RNA in culture supernatants (n = 6) upon treatment with C232A (C) and prostratin (D), respectively. Each dot represents a single donor and data represented is the geometric mean of the values from four replicates per donor. Left y-axis indicates the HIV RNA in cell culture supernatants (blue circles) and right y-axis indicates levels of CD69^+^ CD4^+^ T cells (red triangles). Curve fitting and P values were generated utilizing the 4-parameter-logistics equation in GraphPad Prism.

Using these findings we embarked on new designs for DAG-lactones with additional substituents from the ester moiety in combination with a few olefin substituents. A description of this effort is reported elsewhere [48,49]. Compared with the previously developed compounds, we found that the newly designed molecule, C-232A (Example B10 from WO2020176505 [48] (Fig 1), exhibited even greater selectivity for novel PKC isoforms, as determined by compound-induced PKC translocation in A549 cell lines stably expressing individual PKC isoforms fused to turbo GFP (tGFP) (Table 2). A representative confocal microscopy image for translocation of PKC-θ from cytoplasm to plasma membrane is shown in Fig 4A.

**Table 2:** C-232A exhibits selectivity for novel PKC isoforms.

EC_50_ for PKC isoform translocation in A549-tGFP cells (nM)								
	Classical PKCs				Novel PKCs			
Agonist	PKC-α	PKC-β1	PKC-β2	PKC-γ	PKC-θ	PKC-δ	PKC-ε	PKC-η
C-232A	>39,491	3284	2265	11,510	176	714	58	100
Comin compound 2	>50,000	966	6650	ND	792	4040	783	1194
AJH-863	>50,000	1298	1063	19,390	1202	2316	505	1888

EC_50_, half-maximal effective concentration.

To confirm that C-232A induced translocation of endogenous PKC isoforms, we performed similar experiments with Jurkat cells. Translocation of PKC-θ, but not PKC-α, from the cytoplasm to cell edge was observed after 30 minutes of treatment with C-232A (S3A Fig). As estimated by the ratio F_membrane_/F_cytoplasm_, PKC-θ-specific fluorescence signal increased significantly in the areas near the cell edge upon treatment with C-232A in comparison with DMSO control (P<0.0001), indicating efficient translocation of endogenous PKC-θ to the plasmalemma in the presence of C-232A (S3B Fig).

To further confirm that C-232A can activate endogenous novel PKCs in CD4^+^ T cells, we used cellular fractionation and western blotting after PKC agonist treatment. C-232A translocated PKC-θ from the cytoplasm to the cell membrane in a dose-dependent manner, but did not translocate classical isoforms α, β1, or β2. The nonspecific PKC agonist prostratin translocated both the novel PKC-θ and the classical isoforms PKC-α, PKC-β1, and PKC-β2 with similar potency (S4 Fig). These data further corroborate the improved specificity of C-232A for novel PKC isoforms.

Consistent with these results, the novel and selective PKC agonist C-232A effectively induced HIV expression in total CD4^+^ T cells isolated from ART-suppressed people with HIV. Activation of HIV expression (EC_50_ = 500 nM) correlated with activation of CD69 receptor (early T-cell activation marker) expression (EC_50_ = 57 nM) (Fig 4C), consistent with previous studies indicating that this receptor is a good biomarker for PKC activation [24,29]. Similar correlation was also seen with prostratin (EC_50_ = 2500 and 280 nM) for HIV expression and CD69 activation, respectively (Fig 4D). These results indicate that C-232A, a compound with improved specificity for novel PKC isoforms, activates HIV expression as strongly as less-specific PKC agonists such as prostratin.

### Doses of C-232A required to induce CD69 also cause toxicity in vivo

**Fig 5:**
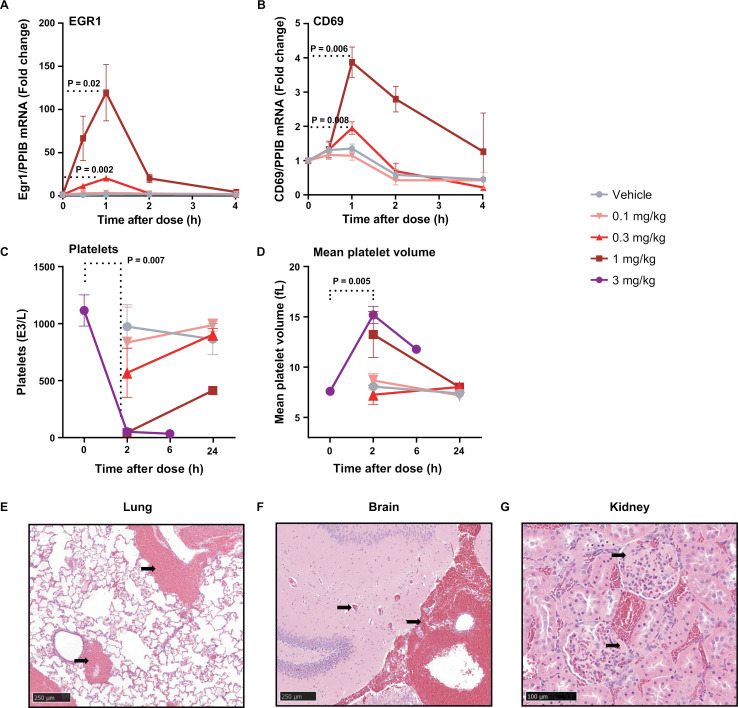
Pharmacodynamic and safety profile of C-232A in vivo in rats. Dose-dependent expression of EGR1 (A) and CD69 (B) mRNA levels measured by QuantiGene analysis in rat whole blood (n = 3) at different time points after C-232A dosing. The y-axis indicates mean fold change in post-dose expression levels compared with predose after normalizing to PPIB mRNA levels. Mean values are plotted with error bars indicating standard deviation. Significant increases are seen in EGR1 levels with 0.3 and 10 mg/kg doses by 1 hour postdose compared with predose levels. P values were calculated using paired *t* test. Rapid (2 hours postdose) dose-dependent changes in circulating platelet levels (C) and mean platelet volume (D) of rats administered C-232A, indicating platelet activation and aggregation. Significant differences between pre-bleed and 2 hours postdose with 3 mg/kg were indicated. P values were calculated using paired *t* test. Representative H&E-stained histopathology images of lung (E), brain (F), and kidney glomeruli (G) from rats dosed with 3 mg/kg C-232A. Arrows in (E&F) indicate hemorrhage and arrows in (G) indicate thrombi within the tissues. Scale bar in (E&F) represents 250 µm. Scale bar in (G) represents 100 µm.

**Fig 6:**
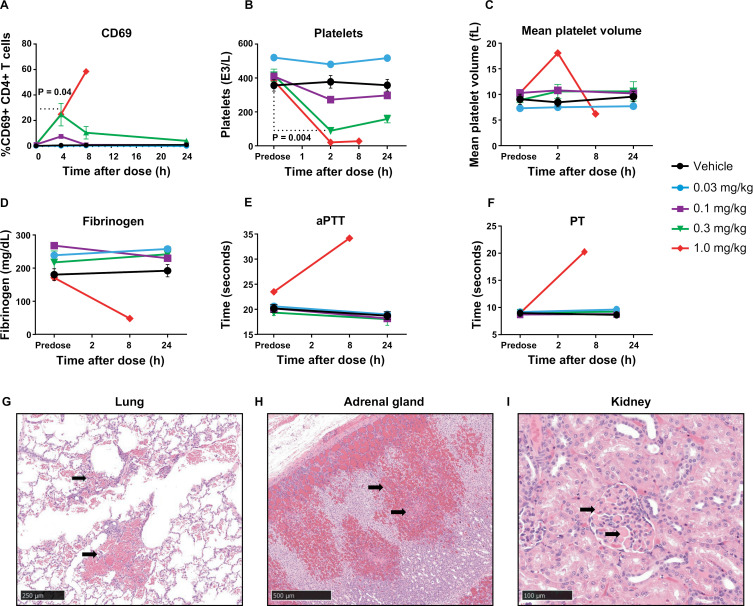
Pharmacodynamic and safety profile of C-232A in vivo in rhesus macaques. (A) Dose-dependent expression of CD69 on CD4^+^ T cells as quantified by flow cytometry in rhesus macaque whole blood at different time points after C-232A dosing (n = 1 for 0.03, 0.1 and 1.0 mg/kg doses; **n** = 3 for 0.3 mg/kg dose and vehicle control). Circulating platelet levels (B) and mean platelet volume (C) upon C-232A dosing are shown. Circulating fibrinogen (D), activated partial thromboplastin time (E), and prothrombin time (F) are shown after administration of C-232A. Statistical significance was calculated using paired *t* tests where appropriate. Representative H&E-stained histopathology images of lung (G), adrenal gland (H), and kidney glomeruli (I) from a rhesus macaque administered 1 mg/kg C-232A. Arrows in (G&H) indicate hemorrhage and in (I) indicate thrombi within the tissues. Scale bar in (G) represents 250 µm. Scale bar in (H) represents 500 µm. Scale bar in (I) represents 100 µm.

C-232A was dosed in rats (Fig 5) and rhesus macaques (Fig 6) to assess pharmacokinetic (PK) and pharmacodynamic properties as well as toxicity. Dosing in Sprague Dawley rats (0.1, 0.3, 1, and 3 mg/kg dose: n = 6 animals/group) (S5A Fig) and rhesus macaques (0.03, 0.1, 0.3, and 1 mg/kg) (S5B Fig) exhibited dose-dependent C-232A PK plasma profiles. Doses of 0.1 and 0.3 mg/kg were well tolerated in all 6 rats dosed. However, 1 of the 6 rats that was administered 1 mg/kg C-232A was euthanized due to severe clinical signs. All rats (n = 6) administered the highest dose (3 mg/kg) were euthanized within 6 hours postdose due to a decline into moribund condition (hypoactivity, cold to touch, labored respiration, and red/purple discoloration). A sentinel dosing approach was implemented for rhesus macaque studies, with only 1 monkey per dose level and dose escalation at least 1 day apart. Doses of 0.03, 0.1, and 0.3 mg/kg were well tolerated. The monkey administered 1 mg/kg of C-232A was euthanized 8 hours postdose due to severe clinical signs (swelling around the eyes, hypoactivity, pallor, and red discolored skin). The 0.3 mg/kg dose of C-232A was well tolerated in 2 additional monkeys, establishing 0.3 mg/kg as the maximum tolerated dose.

Biological activity of C-232A in both rat and rhesus macaque studies was assessed by evaluating CD69, EGR1 (early growth response genes involved in HIV reactivation by PKC agonists) [50,51], and cytokine production. In rats administered C-232A, mRNA expression analysis indicated dose-dependent increases in EGR1 and CD69 transcription (Figs 5A, 5B, S6A and S6B). In monkeys administered C-232A, CD69 expression peaked at 4–8 hours postdose, reaching 59% expression of CD69 on CD4^+^ T cells at the 1 mg/kg dose (Fig 6A). Monkeys administered C-232A also showed elevated cytokine and chemokine levels, consistent with previously reported PKC activation [52,53] (S1 Table).

Clinical pathology parameters were assessed in animals administered C-232A. In both rats and monkeys, circulating platelet counts rapidly decreased and mean platelet volume increased (Figs 5C, 5D, 6B and 6C). These changes were dose dependent and severe at the higher doses of C-232A (<100,000 platelets/µL and >15 fL mean platelet volume). The rapid nature of these changes in platelet parameters suggest platelet activation and aggregation, rather than decreased platelet production [54,55]. In monkeys administered 1 mg/kg C-232A, circulating fibrinogen decreased (Fig 6D) and coagulation times were prolonged (Fig 6E and 6F), consistent with platelet activation and aggregation [56].

To help characterize the toxicity profile of C-232A, tissues from euthanized rats and monkeys were prepared for histopathological evaluation. In both moribund rats and monkeys, widespread thrombi formation and hemorrhage were observed across multiple organs. Representative images show thrombi associated with congestion and hemorrhage in the lung and brain (Fig 5E and 5F) and glomerular thrombi in the kidney (Fig 5G) of rats administered with 3 mg/kg C-232A. In the monkey administered 1 mg/kg C232A, representative images show hemorrhage in the lung and adrenal gland (Fig 6G and 6H) and glomerular thrombi in the kidney (Fig 6I) are shown. These histopathological findings, together with the rapid platelet changes, abnormal coagulation panel, and severe clinical signs, are distinctive hallmarks of DIC.

Given that C-232A induced DIC in rats and monkeys, we tested whether C-232A could directly induce platelet activation and downstream aggregation. Light transmittance aggregometry was conducted in freshly isolated human platelet-rich plasma treated with either C-232A or prostratin. Adenosine diphosphate, a known platelet agonist, was used as a positive control (S7A Fig). Both C-232A and prostratin stimulated platelet aggregation (S7B Fig) in a dose-dependent manner, although C-232A was less potent than prostratin.

### Several classes of PKC agonists induce platelet and CD4^+^ T-cell activation with similar potency

**Fig 7:**
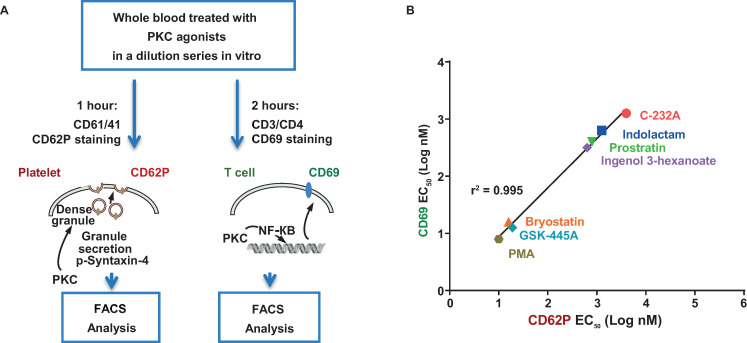
In vitro assay in whole blood predicts PKC agonist platelet toxicity relative to pharmacodynamic potency. (A) Workflow for an in vitro assay that measures platelet (CD62P^+^) and CD4^+^ T-cell (CD69^+^) activation simultaneously in whole blood treated with PKC agonists using flow cytometry. EC_50_ values for activation by several classes of PKC agonists were calculated using 4-parameter, non-linear curve fitting method using GraphPad Prism. (B) Correlation data of several classes of PKC agonists. Y-axis represents CD69 on T cells and x-axis represents CD62P on platelets. Each data point indicates mean EC_50_ value for 2–4 donors for each agonist.

We developed a novel human whole blood assay to determine whether other PKC agonists also activate platelets at the same concentrations that are required to activate CD4^+^ T cells. Platelet activation was assessed by CD62P (P-selectin), which traffics to the cell surface upon activation and α-granule release [57,58]. CD4^+^ T-cell activation was assessed by CD69 upregulation (Fig 7A). All the PKC agonists tested induced platelet activation at concentrations that were similar to those required for T-cell activation (Fig 7B).

### Differential expression analysis indicates PKC-ε and -η isoforms are expressed at high levels in T cells but not in platelets

**Fig 8:**
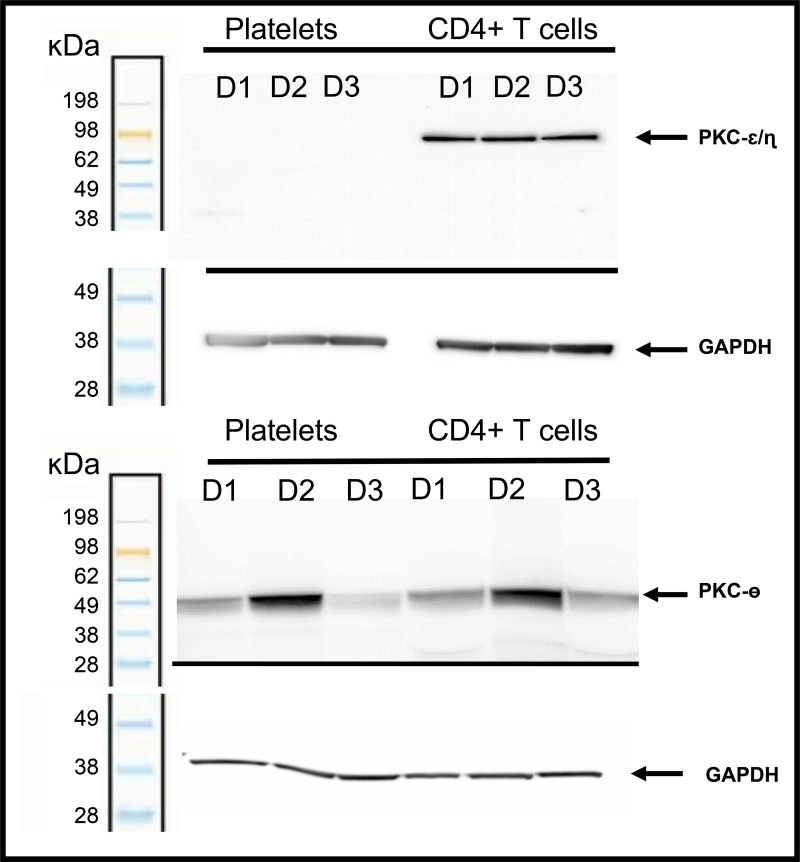
Expression of novel PKC isoforms in human CD4^+^ T cells and platelets. Similar expression of PKC-θ in both T cells and platelets was observed, whereas PKC-η and ε were abundant in T cells but undetected in platelets. Cell lysates of isolated CD4^+^ T cells and platelets from healthy donors (n = 3) were tested for expression of novel PKC isoforms θ, η, and ε by western blotting utilizing PKC-θ and PKC-η/ε antibodies. Expression of GAPDH was used for normalization to enable accurate interpretation of differences in protein expression.

A previous report indicated that PKC-ε and -η are expressed at high levels in T cells but not in platelets [59]. To assess this independently, we isolated CD4^+^ T cells and platelets from healthy individuals (n = 3) and used western blotting to assess PKC isoform abundance in platelets and T cells (Fig 8). To complete this effort, we used the PKC-tGFP A549 cell lines used above to measure PKC translocation to assess the specificity of antibodies reported to bind individual PKC isoforms (S2 Table). We identified antibodies specific for each isoform, with the exception of PKC-ε and -η. For these closely related isoforms, we used an antibody that recognized both isoforms. This analysis showed similar expression of PKC-θ in both T cells and platelets, whereas PKC-ε and -η were found to be abundant in T cells but undetected in platelets. Based on the cross-reactivity of the assessed antibodies, it is also possible that either PKC-ε or -η is not expressed at high levels in T cells. These results indicate that PKC agonists that selectively activate PKC-ε and -η could activate T cells without activating platelets.

### C-233 is a novel PKC agonist with improved PKC-ε selectivity, greater activity in T cells than in platelets, and the ability to activate HIV RNA and p24 expression

**Fig 9:**
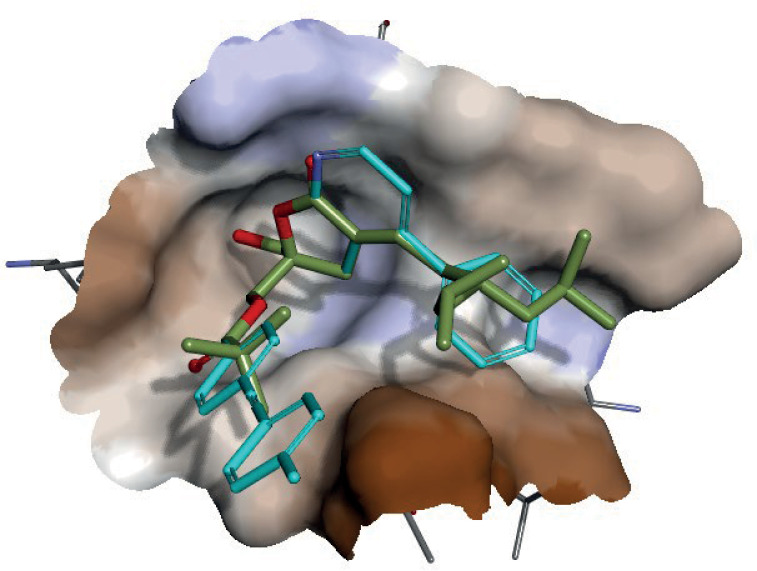
Model of C-233 binding to PKC-δ. C-233 (cyan) was overlayed on compound AJH-863 (green). Note how both the pyridine and the para-phenyl moiety offer multiple ways for analoging in directions pointing into the putative cell membrane direction.

Based on the expression of novel PKC agonists, we hypothesized that a PKC agonist with improved selectivity for PKC-ε would enable activation of T cells with reduced effects on platelets. The branched dimethyl-heptane moiety of C-232A decreases the drug-like properties of the molecule. To improve the drug-likeness of the molecule, the synthetic accessibility, and to enable easier exploration of the dimethyl-heptane binding pocket, we designed another molecule, C-233, based on the structural overlay with C-232A (Fig 9). The exocyclic lactone oxygen and the double bond in C-233 are now wrapped into an isosteric pyridine ring, which can be substituted with an aryl moiety at the para position. In the case of C-233, even a simple phenyl substituent resulted in improved selectivity compared with C-232A (Table 3).

**Table 3:** C-233 exhibits PKC-ε selectivity.

Translocation of novel PKC isoforms in A549-tGFP cells				
	C-232A		C-233	
Isoform	EC_50_ (nM)	ε selectivity	EC_50_ (nM)	ε selectivity
PKC- δ	710	12	47,000	196
PKC-ε	58	1	240	1
PKC-η	100	2	1100	5
PKC-θ	180	3	1500	6

ε selectivity was calculated as EC_50_ of each specific isoform divided by EC_50_ of PKC-ε.

**Fig 10:**
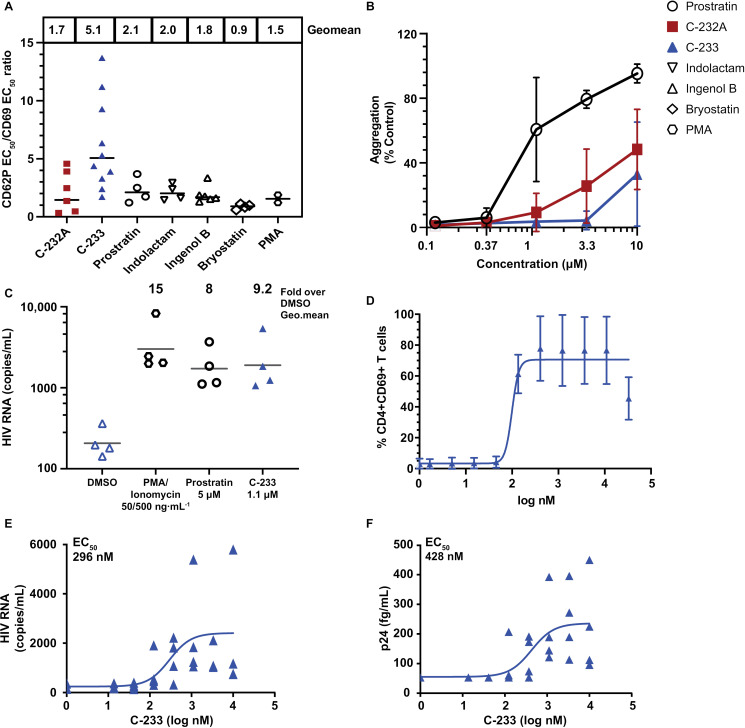
C-233 shows improved potency for T cells in vitro and induces HIV activation ex vivo. (A) Platelet (CD62P^+^) and CD4^+^ T cell activation (CD69^+^) induced by C-233 measured in whole blood from healthy donors (n = 10) by flow cytometry. EC_50_ values calculated using 4-parameter nonlinear regression curve fit. Y-axis shows the ratio of EC_50_ values for platelets to CD4^+^ T cells. Each data point represents a single donor and horizontal bars represent geometric mean. P values were determined by Wilcoxon matched-pair signed rank test. (B) Platelet aggregation induced by PKC agonists, prostratin, C-232A, and C-233, measured by light transmittance aggregometry in purified platelet-rich plasma isolated from healthy donors (workflow in S7 Fig). Each data point represents mean values from all four donors measured at the 20-minute stimulation point with respective compounds in a dilution series as shown. Error bars indicate standard deviation. (C) HIV activation induced in CD4^+^ T cells isolated from ART-suppressed people with HIV is shown for C-233, prostratin and PMA with ionomycin. Each data point indicates geometric mean of 8 replicates from each donor and horizontal lines represent the geometric mean of values from all 4 donors. (D) Total CD4^+^ T cells isolated from people with HIV on ART (n = 2) were treated with C-233 in a dilution series. CD4^+^ T cell activation was assessed by measuring cell surface CD69 expression by flow cytometry. Points indicate the mean and error bars indicate standard deviation. (E) Total CD4^+^ T cells isolated from people with HIV on ART (n = 4) were treated with C-233 in a dilution series and HIV-1 viral RNA induction was measured in culture supernatants at 72 hours after stimulation. Data represented is the geometric mean of the values from all 4 donors and error bars indicate standard deviation. Curve fitting and EC_50_ values were generated utilizing the 4-parameter-logistics equation in GraphPad Prism. (F) p24 levels were measured using an HIV p24 digital immunoassay. Data represented is the mean of the values from all 4 donors (two replicates/donor) and error bars indicate standard deviation. Curve fitting and EC_50_ values were generated utilizing the 4-parameter-logistics equation in GraphPad Prism.

In studies utilizing A549-PKC-tGFP reporter cells (Table 3), C-233 was 5- to 6-fold more potent for PKC-ε translocation compared with PKC-η and PKC-θ, and almost 200-fold more potent than PKC-δ. Based on the low PKC-ε expression in platelets, we predicted that C-233 would be less active in platelets relative to T cells. In whole blood, C-233 (Fig 10A) was 5-fold more potent for T-cell activation relative to platelet activation. This was a 2.4- to 3-fold improvement relative to the other classes of PKC agonists tested. Similarly, in platelet aggregation assays, C-233 was less potent than C-232A and prostratin, with little or no aggregation observed at concentrations below 10 μM (Fig 10B). In peripheral blood mononuclear cells, C-232A and C-233 both induced cytokines associated with PKC activation, consistent with expression of PKC-ε in T cells (S3 Table).

C-233 induced 9-fold activation of supernatant HIV RNA (Fig 10C) after treatment of CD4^+^ T cells from ART-suppressed people with HIV, consistent with the 8-fold activation seen with prostratin in the same donors (n = 6). In CD4^+^ T cells isolated from people with HIV receiving ART, C-233 strongly activated CD69 expression (EC_50_ = 99 nM; Fig 10D). Dose response studies conducted with C-233 showed that activation of HIV RNA occurred at doses similar to those that activated CD69 (Fig 10D and 10E) without impairing viability (S8 Fig). This is consistent with results seen with C-232A and prostratin (Fig 4). HIV protein production was assessed using digital immunoassay to detect p24 in supernatants of CD4^+^ T cells from ART-suppressed people with HIV (Fig 10F) and cellular p24 by the HIV-Flow assay (S9 Fig). Both assays showed that C-233 activated production of viral protein as well as HIV RNA.

### Single dose-ranging study of C-233 in Sprague Dawley rats indicates dose-dependent PK and shows that C-233 is better tolerated than C-232A

**Fig 11:**
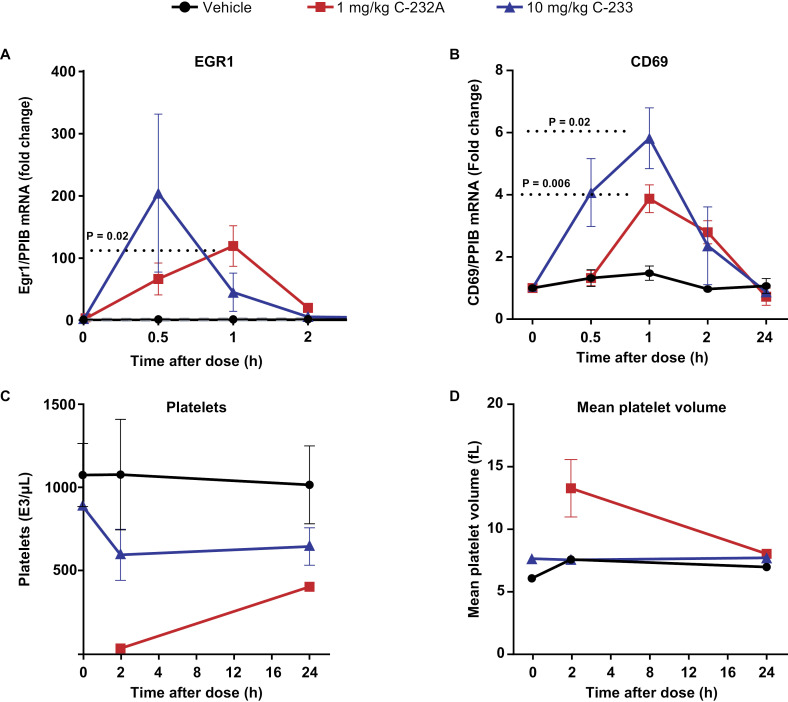
C-233 shows improved tolerability in vivo in comparison to C-232A. Expression levels of (A) Egr1 and (B) CD69 mRNA levels measured by QuantiGene analysis in rat whole blood at different time points after C-233 (10 mg/kg) dosing in comparison with C-232A (1 mg/kg). Expression levels were normalized to predose values after normalizing to PPIB mRNA levels (n = 3). Mean and standard deviation are shown. P values were calculated using paired *t* test. (C, D) Minimal to no changes seen in circulating platelet parameters(C) platelets, and (D) mean platelet volume of rats administered C-233 in comparison with C-232A.

To assess whether improved PKC-ε selectivity is associated with improved tolerability in vivo, ascending concentrations of C-233 were dosed in Sprague Dawley rats. Dose-dependent increases in the PK profile were seen at 1, 3, and 10 mg/kg doses (S10 Fig; n = 3 male rats/group). C-233, at a 10-fold higher dose of 10 mg/kg than C-232A (1 mg/kg), showed higher induction of EGR1 and CD69 mRNA levels (Figs 11A, 11B, S11A and S11B) without any significant depletion of platelets or increased mean platelet volume (Fig 11C and 11D). In addition, while 1 of 6 animals administered 1 mg/kg of C-232A had to be euthanized, no adverse events were observed after dosing 10 mg/kg of C-233. Thus, at doses that activate genes associated with latency reversal, C-233 has reduced platelet toxicity and improved tolerability relative to a less-selective compound.

## Discussion

PKC agonists have been clinically i`nvestigated for the treatment of infectious diseases, cancer, and neurological diseases [10]. However, the advancement of these molecules has been limited by an insufficient understanding of the mechanisms that mediate PKC agonist toxicity in vivo. In this study, we show that PKC agonists cause widespread platelet activation, leading to a syndrome that closely resembles DIC. DIC is characterized by systemic coagulation, resulting in widespread fibrin accumulation and thrombosis within small vessels. This ultimately compromises blood supply to organs, causing organ failure, platelet depletion, and severe bleeding [56]. Consistent with the DIC observed in our studies with rats and monkeys, human platelets are also activated and aggregated after treatment with PKC agonists, as previously reported [60]. The severity of this syndrome makes it a significant concern for the clinical development of PKC agonists. Trials that proceed with PKC agonists should carefully monitor platelet levels, as we found that platelets are depleted at sublethal doses of PKC agonists. We encourage research that pursues approaches to mitigate this toxicity and improve the safety of PKC agonists.

The “shock and kill” strategy targets latent HIV by activating HIV expression and increasing the sensitivity of latently infected cells to immune-mediated pressure. PKC agonists are among the most potent HIV LRAs ex vivo [26]. We confirm the robust HIV activation in our ex vivo studies (Fig 4C and 4D) and extended these findings further to show that multiple novel PKC isoforms are sufficient for HIV activation (S2A Fig). Recent nonhuman primate studies have shown that 15 μg/kg GSK445A, an ingenol-derived PKC agonist, induces latent SIV in an ART-suppressed model of HIV infection [61]. The safety window for GSK445A is narrow, as serious adverse events leading to euthanasia were observed in an animal administered 30 μg/kg [62]. Our findings provide a potential mechanism for the narrow therapeutic window of PKC agonists.

As PKCs are a widely conserved kinase family involved in signaling cascades in many cell types [63], PKC activation could also lead to other undesirable effects. The dose-limiting toxicity for bryostatin is myalgia [64], consistent with described roles for PKC-βII, -ε, and -δ in nociceptor signaling [65–67]. The preclinical models used in our study were not designed to detect pain sensitization and may not have identified adverse effects related to this pathway. PKC agonist activity may also induce significant levels of cytokine production (S1 and S3 Tables) [26,68], which could lead to morbidity at higher doses. Thus, while platelet aggregation is a significant concern for PKC agonists, there are additional pathways modulated by PKCs that could raise further safety concerns.

Several strategies for improving the safety of PKC-mediated activation of latent HIV have been proposed. PKC agonists synergistically activate HIV when combined with other classes of LRAs, such as histone deacetylase inhibitors [25,69]. Combination with a synergistic partner could reduce the PKC agonist dose required for HIV activation, but this has not yet been validated ex vivo or in vivo. Studies of synthetic bryostatin analogs and prodrugs of PKC agonists suggest that modification of the PK profile could improve tolerability [29]. Studies have also been done to identify compounds, such as JAK inhibitors, that decrease cytokine release after PKC treatment without affecting HIV activation [68], but this is unlikely to address potential toxicity related to platelet activation. Finally, selective delivery of PKC agonists to latently infected cells or the cell types that are infected by HIV could improve tolerability. Self-assembling lipid and protein nanoparticles improve PKC agonist delivery to CD4^+^ T cells, but it is likely that greater selectivity will be required [70]. Given the broad expression and function of PKC isoforms, it is likely that specific delivery of PKC agonists to the cell type of interest will be required for well-tolerated HIV activation.

An approach that is complementary to those described above is the selective activation of PKC isoforms required for latent HIV activation. In this study, we show that PKC-ε and -η are sufficient for T-cell activation but have low expression in platelets (Figs 8 and S2B). This suggests that PKC agonists that selectively activate these isoforms could preferentially activate T cells at doses that do not activate platelets. To test this hypothesis, we synthesized a novel PKC agonist, C-233, that had greater selectivity for PKC-ε than previous agonists. This molecule strongly activated HIV expression ex vivo and was more potent for T-cell activation than platelet activation, suggesting an improved tolerability profile (Fig 10). When dosed in vivo, this agonist activated markers associated with HIV activation but did not induce platelet depletion, as the less-selective comparator did (Fig 11). This indicates that selective targeting of PKC-ε can mitigate platelet toxicity without affecting T-cell activation. These results support structure-based design of selective novel PKC agonists for the safe activation of HIV reservoirs.

A similar strategy could be applied to other indications for which PKC agonists could be beneficial. For example, PKC-ε activation could be useful for the treatment of Alzheimer’s disease. Agonists with greater selectivity for PKC-ε than those that have been tested clinically to date could have improved tolerability. A deeper understanding of the isoforms required to achieve the beneficial effects of PKC agonist activity in other indications could facilitate the application of PKC agonists with improved selectivity.

In addition to using selective agonists to avoid platelet activation, it may be helpful to combine PKC agonists with inhibitors that could prevent platelet aggregation. Given the importance of platelet activation in cardiovascular disease, several inhibitors have been developed and validated clinically that reduce platelet activation. However, most of these inhibitors act on proximal events of platelet activation, often affecting the activation of receptors on the platelet surface. PKC enzymes typically function downstream of surface receptors to regulate several platelet functions, including dense and α-granule secretion [71].

Overall, PKC agonists are highly effective at activating latent HIV expression, but their safety profile has been a major hurdle that has prevented the clinical advancement of this entire class. PKC agonist development has been severely limited by a lack of understanding of the mechanism and targets of their adverse effects. PKC isoforms are evolutionarily conserved and broadly involved in multiple critical cellular signaling pathways; therefore, it is essential to properly characterize and address their associated dose-limiting toxicities [72]. Our findings indicate that platelet activation is a major liability associated with currently available PKC agonists. We show that PKC-ε is sufficient for HIV activation and that agonists with increased PKC-ε selectivity have improved in vivo tolerability. Selective PKC isoform activation potentially combined with targeted drug delivery to relevant cell types could significantly alleviate toxicities associated with PKC agonists. This could open new avenues toward more effective and safe HIV latency reversing agents as part of a therapeutic strategy leading to HIV drug-free remission and/or cure.

## Methods

### Ethics statement

People living with HIV and on ART were enrolled at Quest Clinical Research and the AIDS Research Institute, University of California, San Francisco/San Francisco General Hospital and Vitalant in San Francisco, California, USA. Informed written consent was obtained from participants before any study procedures were performed. The study was approved by the Western Institutional Review Board.

All animal work was performed by Covance, Inc. (Madison, Wisconsin, USA). Studies in non-clinical species were conducted at test sites fully accredited by the Association for Assessment and Accreditation of Laboratory Animal Care. The animal protocols and procedures were approved by the Institutional Animal Care and Use Committee (IACUC) at Covance and adhered to the Guide for the Care and Use of Laboratory Animals. All procedures complied with applicable animal welfare acts. An attending laboratory veterinarian was responsible for providing necessary medical treatment to prevent unacceptable pain and suffering in study animals.

### Sex as a biological variable

Our studies exclusively examined male rats and rhesus macaques in an effort to reduce the number of animals used (in adherence to the 3Rs Initiative). It is unknown whether the findings are relevant to female rats or rhesus macaques.

### Statistics

Statistical analysis was performed in GraphPad Prism, version 9.3.0(463) using tests as indicated in the figure legends. Samples treated with PKC agonists were compared with control samples for in vitro and ex vivo experiments. In animal models, predose samples were compared with post-dosing samples. Statistical analysis was done for groups of in vivo studies that contained 3 or more individuals. P values ≤ 0.05 were reported as significant.

### Ex vivo activation of HIV transcription and inhibition by PKC isoform inhibitors

Peripheral blood mononuclear cells and CD4^+^ T cells used for evaluating PKC agonist and inhibitor molecules were isolated from leukopaks of selected participants as described previously [73]. Isolated CD4^+^ T cells (5×10^6^ cells/well) were added to 24-well tissue culture plates in 2.5 mL of RPMI medium containing 100 nM each of elvitegravir and efavirenz. Cells were stimulated with DMSO (vehicle control) or the indicated concentrations of PKC agonist molecules. Cells were pretreated with PKC isoform inhibitors (Selleck Chemicals) for 1 hour before addition of PKC agonist at indicated concentrations and maintained at 37^o^C, 5% CO_2_ for 3 days. Cell supernatants from each well were analyzed using the Cobas AmpliPrep/AmpliTaq system for quantification of HIV-1 RNA using the HIV-1 test v2.0 kit (Roche Diagnostics).

### CD4^+^ T-cell activation flow cytometry

Sixteen hours post treatment with PKC agonists, cellular activation of 100,000 CD4^+^ T cells, taken from the above ex vivo HIV activation cultures, was assessed using flow cytometry. Cell surface expression of CD69 was measured on CD4^+^ T cells using anti-human CD3 (UCHT1), CD4 (SK3) and CD69 (FN50) antibodies and standard flow staining methods. Stained cells were acquired on the Becton Dickinson (BD) LSR Fortessa II flow cytometer and results were analyzed using FlowJo software.

### Ex vivo HIV-Flow assay in CD4^+^ T cells

Isolated CD4^+^ T cells from ART-suppressed people with HIV were cultured in RPMI medium containing 10% FBS, 100 nM efavirenz, and 1 μM tenofovir alafenamide at 2 million cells per mL. These cells were subsequently stimulated with DMSO (vehicle control), 500 nM PKC agonist C-233, or 162 nM PMA and 1 µg/mL ionomycin. The cells were cultured at 37°C, 5% CO_2_ for 3 days. After 3 days, the cells were washed with PBS, stained with Live/Dead Fixable Aqua for 25 minutes at 4°C, followed by 2% FBS in PBS wash. Cells were then fixed and permeabilized in FoxP3 Fix/Perm buffer (E-Bioscience, catalog 005523) for 45 minutes at 4°C, followed by FoxP3 Perm buffer wash. The samples were stained with 2 different anti-p24 antibodies, (KC57-PE/ Beckman Coulter, 6604667, and a28B7-APC Medimabs, MM-0289-APC) diluted 1:1000 in FoxP3 Perm buffer for 45 minutes at 4°C, washed with FoxP3 Perm buffer, and resuspended in PBS. The samples were run on the BD LSR Fortessa II flow cytometer. The data were analyzed using FlowJo version 10.7.1.

### In vitro whole blood platelet and CD4^+^ T-cell activation assay

Donors were required to withdraw from nonsteroidal anti-inflammatory drugs, dietary supplements, and birth control for 48 hours before blood collection. Whole blood was drawn into sodium citrate tubes using a 21-gauge needle. To prevent potential interference from tissue thromboplastin released during venipuncture [74], which could affect the accuracy of coagulation testing, the first 2 mL of blood drawn was discarded. PKC agonist molecules were dispensed in a dilution series into 80 μL of blood per well in a flat-bottom 96-well plate and mixed gently. Care was taken not to introduce any mechanical stress-related activation of platelets. After 1 hour at 37ºC, 5% CO_2_, samples were mixed and 8 μL was transferred to a second plate with 72 μL of platelet buffer (1 M HEPES, 5 M NaCl, 2 M KCl, 1 M MgCl_2_, 12 mM NaHCO_3_, 0.4 mM Na_2_HPO_4_, 5.5 mM anhydrous D-glucose, 0.2 μM filter sterilized and stored at 4ºC up to a month; BSA was added to 0.35% at the time of use to the required amount) per well. The samples were then stained with 1 μL of antihuman CD41/61 (clone A2a9/6) and antihuman CD62P (clone AC1.2) along with 38 μL of platelet buffer and was incubated in the dark for 30 minutes. 100 μL of BD Phosflow buffer (BD Biosciences, catalog 557870) was added to the samples, mixed gently, and 50 μL was acquired on BD LSR Fortessa II at 0.5 μL/second flow rate to measure platelet activation. The remaining 72 μL of blood in the first plate was incubated for 1 additional hour and stained with anti-human CD3 (UCHT1), CD4 (SK3), and CD69 (FN50) (30 minutes in the dark). Red blood cell lysis and fixing was done using BD lyse/fix solution. Stained cells were acquired on BD LSR Fortessa II after washing. Data were analyzed using FlowJo software version 10.7.1. EC_50_ values for platelet and CD4 T-cell activation for each agonist was obtained by nonlinear regression curve fit using GraphPad Prism version 9.3.0.

### PKC isoform translocation assay

Cellular translocation of PKC isoforms upon activation was determined in 8 A549 cell lines, each expressing a single PKC isoform fused to tGFP (PhenoVista Biosciences). Cells were plated in 384-well plates (80 μL/well) at 10^5^ cells/mL in RPMI with 10% FBS and rested overnight. PKC agonist(s) were serially diluted at 1:3 steps in DMSO and added to the cells. After 60 minutes in an incubator at 37^o^C, 5% CO_2_, cells were stained with Hoechst-33342 and fixed with 2% paraformaldehyde for 30 minutes at 4^o^C. Cells were then stained with wheat germ agglutinin conjugated to AlexaFluor647 (WGA-647) for 10 minutes at 4^o^C, washed to remove excess dye and imaged with Cellomics Array Scan (Thermo Fisher) at room temperature (RT). Images were analyzed using Cellomics automated image analysis algorithm. Plasma membrane translocation was quantified using the ratio between the average fluorescence in the areas at the cell edge defined by WGA-647 and the cytoplasm region.

### Isoform sufficiency studies for HIV activation

Sufficiency of novel PKC isoforms for HIV activation was studied using 2 cell line systems. In the first system, latent HIV Jurkat (A05) reporter cell lines were generated by acute infection of Jurkat WT A05 cells with HIV-1 NL4-3 Gag-iGFP ΔEnv, which were initially rested and sorted on the GFP negative population. Sorted cells were then activated with 5 μM prostratin for 48 hours and sorted on GFP positive cells. To study reactivation of latent cells by various PKC isoforms, 30 µg of pcDNA3.1 RFP vectors containing either PKC-ε, -η, -θ, or -δ, were transfected (GenScript’s Neon transfection system) according to the manufacturer’s instructions [38]. PRKCH A160E T2a RFP (accession number: NM-006255.4) was cloned using NotI/Xba restriction site digestion, PRKCQ A148E T2a RFP (accession number: NM.006257.4), PKCE A159E T2a RFP (accession number: NM_005400.2), and PRKCD RFP deletion 1.333 T2a RFP (accession number: NM_001354676) were all cloned using BamHI/NotI restriction site digestion. DNA was isolated from transformed *Escherichia coli* and sequences were confirmed before transfections. Percent RFP+ and GFP+ cells were measured by flow cytometry after 48 hours in culture. Double positive cells were measured as cells expressing both constitutively active PKC isoform (RFP+) and reactivated latent HIV (GFP+). Prostratin was used as a positive control, in which case only GFP+ cells were taken as cells with HIV reactivation. In the second HEK293-luciferase reporter cell system, constitutively active novel PKC isoform constructs (same pcDNA3.1 RFP vectors that were used in the latent Jurkat experiments) were transfected using Lipofectamine 2000 in NF-kB reporter (Luc) – HEK293 (BPS Bioscience, catalog 60650) and AP1 reporter (Luc) – HEK293 (BPS Bioscience, catalog 60405) cell lines. TNFα (Sigma, catalog T0157-10UG) and phorbol 12-myristate 13-acetate (LC Laboratories, catalog P-1680) were used as positive controls for NF-kB and AP1 pathways, respectively. Transfected cells were incubated for 48 hours, and luciferase activity was measured per manufacturer’s instructions using the One-Step Luciferase Assay System (BPS Bioscience, catalog 60690).

### In vivo rat studies

Wild-type male Sprague Dawley (Hsd: Sprague Dawley SD) rats (8–15 weeks old) were sourced from the Covance stock colony for conduct of in vivo studies at Covance Laboratories Inc. (Madison, Wisconsin, USA). Rats were administered a single dose of vehicle control (n = 3), or 0.1, 0.3, 1, or 3 mg/kg C-232A (n = 6), or 10 mg/kg C-233 by intravenous infusion over 30 minutes. Blood samples were collected through 24 hours postdose. Samples for PK measurement of C-232A concentration were processed to plasma and shipped to Gilead Sciences (Foster City, California, USA). Samples were analyzed at Covance Laboratories for hematology and clinical chemistry panels. At unscheduled necropsy (3 mg/kg C-232A), tissues were preserved in 10% neutral-buffered formalin, embedded in paraffin, mounted on slides, and stained with hematoxylin and eosin. Tissues were then evaluated independently (Experimental Pathology Laboratories, Inc).

### In vivo monkey studies

Male rhesus macaque monkeys (young adult to adult) were sourced from the Covance stock colony for conduct of in vivo studies at Covance Laboratories Inc. (Madison, Wisconsin, USA). Monkeys were administered a single dose of vehicle control (n = 3), 0.03, 0.1, 0.3, or 1 mg/kg of C-232A (n = 1) by intravenous infusion over 30 minutes using a sentinel approach. An additional 2 male monkeys were administered 0.3 mg/kg C232A after it was determined that this was the maximum tolerated dose level. Blood samples were collected through 24 hours postdose. Samples for PK (for measurement of C-232A concentration) and cytokine analysis were processed to plasma and shipped to Gilead Sciences. Activation of CD4^+^ T cells was assessed by measuring CD69 expression levels in whole blood by flow cytometry (Covance Laboratories). Samples for clinical pathology were analyzed at Covance Laboratories for hematology, coagulation, and clinical chemistry panels. At unscheduled necropsy (1 mg/kg C-232A), tissues were preserved in 10% neutral-buffered formalin, embedded in paraffin, and processed to slides for staining with hematoxylin and eosin. Tissues were then evaluated independently (Experimental Pathology Laboratories, Inc).

### Bioanalysis of C-232A and C-233 in rat and rhesus plasma

Rat and rhesus plasma samples (20 µL aliquot of each) were treated with 100 µL acetonitrile containing a generic internal small-molecule standard. After precipitation of the protein component, 100 µL of the supernatant was transferred to a clean 96-well plate and run through an API-5000 triple quadrupole mass spectrometer (SCIEX, Framingham, Massachusetts, USA) with electrospray ionization in positive mode. The descriptive PK parameters were analyzed using noncompartmental analysis with Phoenix WinNonlin software (version 8.2, Certara USA, Inc.). The area under the plasma concentration–time curve (AUC_last_) values were calculated using the linear trapezoid method.

### Measurement of EGR1 and CD69 mRNA levels in rat blood

EGR1 and CD69 mRNA expression levels were measured in whole blood, processed using Affymetrix QuantiGene Sample Processing Kit (Thermo Fisher Scientific, catalog QS0111). Quantigene multiplex analysis was performed on 1:3 diluted blood using probes specific for rat EGR1 (Thermo Fisher Scientific, catalog nm_012551) and CD69 (catalog nm_134327), as per the kit’s instructions (Thermo Fisher Scientific, catalog QP-1014) and normalized to PPIB (catalog nm_022536) [75] and β-actin (catalog nm_031144).

### Measurement of CD69 expression levels in rhesus blood

CD69 protein expression levels in whole blood were measured using flow cytometry. Samples, collected in K2EDTA tubes, were surface stained with anti-human monoclonal antibodies against markers CD3 (UCHT1), CD4 (SK3), and activation marker CD69 (FN50) for 30 minutes at RT. Red blood cells were lysed and cells were fixed (BD Lyse/Fix buffer) according to the manufacturer’s instructions. All data were acquired on an LSR II (BD Biosciences) and were analyzed using FlowJo software.

### Cytokine/chemokine analysis in plasma and cell culture supernatants

For plasma cytokine analysis (S1 Table), whole blood was collected into K2EDTA tubes from C-232A and vehicle dosed rhesus macaques (prebleed and various post-dose time points) and plasma was isolated and stored at −80^º^C. Cytokine/chemokine analysis in plasma samples was accomplished using a nonhuman primate-specific 37 Plex ProcartaPlex immunoassay system (Thermo Fisher Scientific) according to the manufacturer’s instructions. Samples were then read on a Luminex 200 (Luminex) platform and analyzed using Bio-plex Manager software (Bio-Rad).

For in vitro cytokine analysis (S3 Table), cryopreserved, healthy human PBMCs (n = 5 donors, n = 3 triplicate wells/donor) were treated with various concentrations (0.6 to 10000 nM) of either prostratin, C-232A, or C-233 for 24 hours. Culture supernatants were analyzed for induction of several cytokines as shown in S3 Table, using a 10-plex MSD (Meso Scale Discovery) assay following manufacturer’s instructions. MSD Workbench 4.0 software was used for data analysis and 4-parameter logistic curve fit model was utilized to determine values of experimental samples from standard curves. Values for each cytokine (pg/mL) for a given concentration was calculated as the geometric mean of all triplicate values combined from all 5 donors.

### Crystallization and structure determination

Purified protein (PKC-δ C1B domain) was concentrated to 5 mg/mL and crystallized at 20°C using the sitting-drop vapor-diffusion method by mixing 100 nL of the concentrated protein with 100 nL of the reservoir solution containing 10% PEG 3350 and equilibrated against 100 µL of the reservoir solution containing 30% PEG 3350. The apo C1B crystals, once formed, were soaked overnight in a cryoprotectant solution and then flash frozen in liquid nitrogen for X-ray diffraction studies.

X-ray diffraction data were collected at a wavelength of 1.2 Å at the Advanced Light Source, Beamline 5.0.1 (Berkeley, California, USA). The protein-agonist complex crystallized in space group C222_1_ with cell parameters: a = 63.3 Å, b = 65.7 Å, c = 32.5 Å, α = 90.0°, β = 90.0°, and γ = 90.0°. The data were processed and scaled with the program XDS. Initial models were obtained by molecular replacement using the EPMR program version 16.07.1 and utilizing coordinates from a single monomer of a previously determined complex of PKCδ C1B domain in complex with an agonist [PDB id: 1PTR] (with coordinates for the agonist atoms removed) as the search model. The initial model was further refined using multiple rounds of simulated annealing in the Phenix software package followed by manual refitting of the model in COOT. In the later stages of refinement, strong residual electron density in the agonist binding site allowed for unambiguous placement of AJH-863 in the structure followed by final refinement of the model.

### Structure-based design of C-233

Compounds were modeled into our crystal structure of PKC-δ C1B domain described above using the Schrodinger software package using the MacroModel EMBRACE module.

### Confocal microscopy to study translocation of PKC isoforms

A549 cells expressing tGFP-labeled PKC isoforms were plated in an 8-chamber cell imaging cover glass (Eppendorf, catalog 0030742036) and cultured in RPMI medium supplemented with 10% heat-inactivated FBS, antibiotic-antimycotic agent (Gibco-BRL, catalog 15240-062) antimycoplasma agent, Plasmocin (InvivoGen, catalog ant-mpt-1). Cells were pretreated with Hoechst 3342 for nuclear staining and then imaged upon addition of the compounds at 37ºC to assess localization of tGFP-labeled proteins using confocal laser scanning microscopy with a Leica SP8 (Leica Microsystems Inc., Wetzlar, Germany). Activation of PKC isoforms was assessed by changes in their intracellular localization.

### Immunofluorescence of endogenous PKC-θ and PKC-α in Jurkat cells

Cells were cultured in RPMI medium supplemented with 10% heat-inactivated FBS and penicillin-streptomycin. For treatment, cells were incubated in either DMSO or C-232A for 30 minutes at 37^o^C. Cells were fixed in ice-cold methanol, washed, and blocked with 2% BSA for 60 minutes at RT. Blocked cells were stained with either a 5 μg/mL primary PKC-θ antibody (R&D Systems, catalog MAB4368) or a 1:100 primary PKC-α antibody (R&D Systems, catalog MAB5340SP) in PBS with 1% BSA at 22^o^C for 90 minutes. Donkey anti-mouse IgG antibody conjugated with Alexa Fluor 647 (Thermo Fisher Scientific, catalog A-31571) at 2 μg/mL and goat anti-rat IgG H&L antibody conjugated with Alexa Fluor 488 (Abcam, catalog ab150157) were used as secondary antibodies, respectively, in PBS with 1% BSA at 22^o^C for 60 minutes. After immunostaining, the cell suspension was mixed (1:1) with ProLong Gold antifade reagent containing DAPI (Life Technologies, catalog P36935) and 5 μL of the mix was applied onto a microscope slide, covered with a coverslip, and cured overnight in the dark at RT. Samples were imaged with a Leica SP8 confocal microscope. Image analysis was performed using ImageJ software.

### Platelet isolation

8.5 mL of fresh whole blood from healthy donors (n = 3) was collected in 10 mL acid citrate dextrose (ACD) tubes containing 1.5 mL of ACD solution (4 tubes/donor). Blood was centrifuged at 150×g for 30 minutes to obtain platelet-rich plasma (PRP). 4 mL of PRP (top 2 mL of the PRP from each tube) was pooled into a 15-mL conical tube, mixed gently with HEPES buffer at a 1:1 ratio, and centrifuged at 100×g for 20 minutes. 7 mL of the supernatant was transferred into a separate 15-mL tube and centrifuged again to yield purified PRP. 2 mL aliquots of purified PRP were transferred into new tubes and centrifuged at 800×g for 15 minutes to pellet the platelets. Supernatant was discarded and platelets were stored at −80ºC. Platelets from 1 aliquot were resuspended very gently in 200–300 μL of platelet buffer to measure purity, quantity, and activation by flow cytometry. 100 μL of resuspended platelets was stained with 2 μL each of anti-human-CD61/41 (Biolegend, clone A2a9/6) and anti-human CD62P (BD Biosciences, Clone AC1.2) for 30 minutes at RT. After staining, 200 μL of Phosflow buffer (BD Biosciences, catalog 557870) and 50 μL of CountBright absolute counting beads (Invitrogen, catalog C36950) were added and data were acquired on the LSR Fortessa II with 1000 events gated on beads. Samples were run at the slowest possible speed (0.5 μL/second) to avoid platelet scatter. Absolute counts of platelets were calculated by comparing the ratio of bead events to cell events.

### Western blotting

Total protein cell lysates were extracted from isolated CD4 T cells and platelets of 3 healthy donors using RIPA buffer (Thermo Fisher Scientific, catalog 89900), and protein concentrations quantified using Pierce BCA assay (Thermo Fisher Scientific, catalog 23227). Lysates were prepared for SDS PAGE by addition of NUPAGE sample reducing agent (Life Technologies, catalog NP0004) and NUPAGE LDS sample buffer (Life Technologies, catalog NP0007) and heated to 95°C for 5 minutes. 50 µg of total protein was fractionated on a 10% Bis-Tris midi protein gel (Invitrogen, catalog WG1201BOX) for 1.5 hours followed by transfer onto PVDF membrane (Invitrogen, catalog IB401001) using an iBLOT instrument. For experiments shown in Fig 8, primary antibodies against PKC-ɛ and -ɳ (LSBio, catalog C172661, 1:2000 dilution), PKC-θ (Cell Signaling Technology, catalog 13643, 1:1000 dilution), and GAPDH (Cell Signaling Technology, catalog 5174S, 1:1000 dilution) at RT for 60 minutes and secondary antibodies rabbit anti-mouse IgG-HRP (Santa Cruz Biotech, catalog sc-358914, 1:2000) and anti-rabbit IgG-HRP (Cell Signaling Technology, catalog 7074, 1:2000 dilution) at RT for 60 minutes were used. Membranes were developed using an Azure Biosystems c600 instrument with SuperSignal West Dura extended-duration pro-luminescent substrate (Invitrogen, catalog 37071).

### Cell fractionation

CD4^+^ T cells treated with C-232A for 1 hour in a concentration-dependent manner were separated into cytoplasmic and membrane protein fractions using a cell fractionation kit (Cell Signaling Technology, catalog 9038) and protein concentrations quantified using a Pierce BCA assay (Thermo Fisher Scientific, catalog 23227). 4–6 μg of fractionated total protein lysate was fractionated on SDS-PAGE gel using Peggy Sue (Protein Simple). The resulting area under the curve was normalized against β-actin.

### Platelet aggregation assay

Human PRP was isolated from healthy donor blood (n = 3) and treated with C-232A, C-233 and prostratin in a dose-dilution curve. Aggregation of platelets was assessed using light transmittance aggregometry using standard methods [76].

### HIV p24 digital immunoassay

The fully automated Quanterix HD-X analyzer for single molecule detection was utilized to measure HIV p24 levels in culture supernatants (stored frozen at –80°C until use) [77,78]. Upon thaw, Triton X-100 was added to a final concentration of 1% to culture supernatants, incubated for 1 hour at room temperature before analysis on the same day. An in-house standard using purified HIV p24 protein was utilized in place of the standard provided in the p24 Simoa kit (Quanterix, catalog 102215). Briefly, p24 protein purchased from Advanced Biotechnologies, catalog 14-101-050, was reconstituted to 200 ng/mL in complete RPMI and stored at –80 in smaller aliquots. Standard curve was made in a 3.5-fold dilution series from 40000 to 1.8 fg/ml in complete RPMI with 1% Triton X-100. All other assay reagents and assay reaction conditions followed the manufacturer’s protocol. p24 concentration in experimental samples was calculated based on a p24 calibration curve with 4-parameter curve fitting.

## Supporting information

S1 Table Cytokine levels in plasma at 4 hours post C-232A dose in rhesus macaques. (PDF)

S2 Table PKC isoforms antibody screen. Cross reactivity (+), no cross reactivity (-); *Antibody utilized in western blot experiment in Fig 8.
(PDF)

S3 Table Cytokine induction in vitro in PBMC culture supernatants treated with non-selective and selective PKC agonists. (PDF)

S1 Fig HIV activation by novel PKC isoforms. Figure representing untransformed HIV RNA values from Fig 2. HIV viral RNA (copies/mL) in total CD4^+^ T cells from ART-suppressed people with HIV (n = 12) treated with PKC agonists (prostratin [red circles, n = 7]/Comin compound 2 [red triangles, n = 5]) alone, with pan-PKC inhibitor Gӧ-6850 (green circles/green triangles), or with classical PKC inhibitor Gӧ-6976 (blue circles/blue triangles). PMA and ionomycin (black diamonds) were used as positive control. DMSO (vehicle) was used as negative control (black circles and green/blue squares). Each symbol represents the geometric mean for a single individual (n = 3 to 8 replicate wells). Horizontal bars indicate the geometric means of all individuals in the indicated condition.
(PNG)

S2 Fig Sufficiency of individual novel PKC isoforms for activating HIV expression. (A–B) HIV and CD69 activation in latent Jurkat latent reporter cell line expressing constitutively active PKC-ε, η, θ, and δ. Percent HIV expression was measured in Jurkat cell line latently infected with HIV-1 NL4-3 Gag-iGFP Δenv and transiently transfected with constitutively active novel PKC isoforms ε, η, θ or δ utilizing pcDNA3.1. Expression was measured at 48 hours post transfection by gating on GFP+ (positive for HIV activation) and RFP+ (positive for transfected and PKC isoform expressing) cells. Prostratin was a positive control representing cells treated with 0.4 μM prostratin for 48 hours alone without any transfection. (A) The percent HIV activation obtained under each condition. (B) Percent CD69 expression at 48 hours in the same cells indicating activation upon expression of constitutively active novel PKC isoforms. Expression of luciferase driven by promoters responsive to NF-kB (C) and AP-1 (D) in HEK293 cells expressing constitutively active novel PKC isoforms. Transfected cells were incubated for 48 hours, and luciferase activity was measured using One-Step Luciferase Assay.
(PNG)

S3 Fig Activation of endogenous PKC-θ but not PKC-α in Jurkat cells treated with C-232A. (A) Confocal images of immunostained Jurkat cells showing translocation of endogenous PKC-θ (bottom panel/right) from cytoplasm to cell membrane upon 30-minute treatment with C-232A but not PKC-α (bottom panel, middle). Clear translocation of PKC-θ from cytoplasm to cell edge can be seen despite the small distance between plasma membrane and nuclear envelope in C-232A treated samples. Cells treated with vehicle control, DMSO, do not show translocation of either of these isoforms (top, right and middle). DAPI staining indicates (left panels) nuclear location within each cell. (B) Translocation efficiency of PKC isoforms was quantified using the ratio between the average fluorescence at the cell edge versus the cytoplasmic region. Significant increase in this ratio is seen for PKC-θ in cells treated with C-232A (red fluorescence) compared with PKC-α (green fluorescence), suggesting that C-232A effectively activated and translocated novel isoform PKC-θ but not PKC-α. Cells treated with vehicle control, DMSO, do not show translocation of either of these isoforms. Each individual data point represents the ratio of fluorescent signal from individual cells that were analyzed. Horizontal bars represent geometrical means with standard deviation and P values were obtained using Mann–Whitney test.
(PNG)

S4 Fig Translocation of PKC isoforms in primary CD4^+^ T cells by C-232A. (A) Western blot of PKC isoforms θ, α, β2, and β1 in cytoplasmic and membrane fractions of CD4^+^ T cells treated with C-232A. Quantitative measurement of translocation of PKC isoforms by C-232A (B) and prostratin (C) in CD4^+^ T cells. Area under the peak generated by western blot analysis of membrane-associated PKC isoforms after treatment and plotted using GraphPad software. AUC plots were used to generate EC_50_ values (D).
(PNG)

S5 Fig PK profile of C -232A in rats and rhesus macaques. (A) Plasma concentrations of C-232A from dose escalation studies in Sprague Dawley rats, dosed by slow IV infusion. Rats were dosed at 0.1, 0.3, 1, and 3 mg/kg and plasma concentrations of C-232A were measured over a time period of 24 hours after dosing. (B) Plasma concentrations of C-232A from dose escalation studies in rhesus macaques, dosed by slow, IV infusion. Rhesus were dosed at 0.03, 0.1, 0.3, and 1 mg/kg and plasma concentrations of C-232A were measured over a time period of 24 hours after dosing.
(PNG)

S6 Fig Additional analysis for demonstration of pharmacodynamics of C-232A (Fig 5A and 5B) utilizing β-actin as an internal PCR control gene. Dose-dependent expression of EGR1 (A) and CD69 (B) mRNA levels measured by QuantiGene analysis in rat whole blood (n = 3) at different time points after C-232A dosing. The y-axis indicates mean fold change in post-dose expression levels compared with pre-dose after normalizing to β-actin mRNA levels. Mean values are plotted with error bars indicating standard deviation. P values were calculated using paired *t* test.
(PNG)

S7 Fig C-232A and prostratin induce platelet aggregation. (A) Assay principle for platelet aggregation assay. Platelet rich plasma made from fresh whole blood from healthy donors (n = 3) was treated with either C-232A or prostratin in a dilution series for 20 minutes and rate of light transmission before and after treatment was measured by light transmittance aggregometry. (B) In vitro platelet aggregation caused by prostratin (black open circles), C-232A (red squares), and an assay positive control (adenosine diphosphate [ADP]; (brown circles).
(PNG)

S8 Fig Effect of C-233 on cell viability. CD4^+^ T cells from ART-suppressed people with HIV were treated with the indicated C-233 concentrations for 72 hours. Cell viability was assessed using Promega Cell Titer Glo according to manufacturer’s instructions. Bars indicate the means and lines indicate standard deviation from 4 donors, each tested with 8 replicates.
(PNG)

S9 Fig C-233 induces HIV activation in CD4^+^ T cells from ART-suppressed people with HIV. Dot plots showing HIV-Flow anti-p24-PE (KC57) and anti-p24-APC (28B7) antibody co-staining in CD4^+^ T cells isolated from 2 ART-suppressed individuals with HIV treated with DMSO (control), 500 nM C-233, or 162 nM PMA and 1 µg/mL ionomycin for 72 hours. The indicated gates define the p24 double positive (p24 DP) population of reactivated HIV-infected cells.
(PNG)

S10 Fig PK profile of C-233 in rats. Plasma concentrations of C-233 from dose escalation studies in Sprague Dawley rats, dosed by slow IV infusion. Rats (n = 3) were dosed at 1, 3, and 10 mg/kg and plasma concentrations of C-233 were measured for 24 hours after dosing. After in vivo administration, dose-dependent concentrations of C-233 in plasma were noted.
(PNG)

S11 Fig Additional analysis for demonstration of pharmacodynamics of C-233 (Fig 11A and 11B) utilizing β-actin as an internal PCR control gene. Expression levels of (A) Egr1 and (B) CD69 mRNA levels measured by QuantiGene analysis in rat whole blood at different time points after C-233 (10 mg/kg) dosing in comparison with C-232A (1 mg/kg). Expression levels were normalized to predose values after normalizing to β-actin mRNA levels (n = 3).
(PNG)
