# Supplementary material for: Activating PKC-ε induces HIV expression with improved tolerability
Source: PLoS Pathog. 2025 Feb 6;21(2):e1012874. doi: 10.1371/journal.ppat.1012874 (PMC11801715; doi:10.1371/journal.ppat.1012874)
Supplement: S1 Table — (PDF) [file ppat.1012874.s001.pdf]

Table S1. Cytokine levels in plasma at 4 hours post C-232A dose in rhesus macaques.

Cytokine (pg/mL)	Vehicle	0.03 mg/kg	0.1 mg/kg	0.3 mg/kg	1 mg/kg
IL-6	87	43	126	76	3872
IL-8	216	275	2531	375	6153
IL-1β	43	45	70	59	343
IL-1RA	117	169	775	2265	4471
MIP-1α	228	208	160	187	1111
MIP-1β	183	131	428	707	3913
I-TAC	112	88	427	365	5602
MIG	89	55	64	55	658
MCP-1	756	1900	3250	6117	13,620
VEGF-A	160	107	161	81	330
IL-18	165	114	193	145	351
IFN-γ	54	43	75	53	200
IFN-α	166	90	143	112	187
TNF-α	48	56	39	19	28
IL-10	88	42	80	54	82
