# Supplementary material for: Activating PKC-ε induces HIV expression with improved tolerability
Source: PLoS Pathog. 2025 Feb 6;21(2):e1012874. doi: 10.1371/journal.ppat.1012874 (PMC11801715; doi:10.1371/journal.ppat.1012874)
Supplement: S2 Table — Cross reactivity (+), no cross reactivity (-); *Antibody utilized in western blot experiment in Fig 8. (PDF) [file ppat.1012874.s002.pdf]

Table S2. PKC isoforms antibody screen.

PKC antibody	PKC isoform cross-reactivity								Catalog #	Clone #	Vendor
**Reported specificity**	**α**	**β1**	**β2**	**ɵ**	**δ**	**ε**	** η**	**γ**			
PKC-α	+	-	-	-	-	-	-	-	sc-8393*	H-7	Santa Cruz Biotechnology
PKC-β1	-	-	-	-	-	-	-	-	ab195039	EPR18512	Abcam
PKC-β1	-	+	-	-	-	-	-	-	sc-8049	E-3	Santa Cruz Biotechnology
PKC-β2	-	-	+	-	-	-	-	-	sc-13149	F-7	Santa Cruz Biotechnology
PKC-θ	-	-	-	+	-	-	-	-	13643	E1I7Y	Cell Signaling Technology
PKC-δ	-	-	-	-	+	-	-	-	9616S	D10E2	Cell Signaling Technology
PKC-η	-	-	-	-	-	-	-	-	NSB969	p Thr655	Novus Biologicals
PKC-η	-	-	-	-	-	-	-	-	ab179524	EPR18513	Abcam
PKC-ϒ	-	-	-	-	-	-	-	-	sc-166385	C-4	Santa Cruz Biotechnology
PKC-ε	-	-	-	-	-	+	+	-	LS-C172661	OTI1B7	LS Bio
PKC-ε	-	-	-	-	-	+	+	-	LS-C357512	NA	LS Bio
PKC-ε	-	-	-	-	-	+	+	-	LS-C172682	OTI2A12	LS Bio
PKC-ε	-	-	-	-	-	+	+	-	LS-C172684	OTI4B3	LS Bio
PKC-ε	-	-	-	-	-	+	+	-	LS-C172683	OTI2E9	LS Bio
PKC-ε	-	-	-	-	-	+	+	-	LS-C172674	OTI1B4	LS Bio
PKC-ε	-	-	-	-	+	+	-	-	2683	22B10	Cell Signaling Technology
PKC-ε	-	-	-	-	+	+	-	-	610085	21/PKC ε	BD Transduction

PKC-ε	-	-	-	-	+	+	-	-	20877	NA	Protein Tech
PKC-ε	-	-	-	-	+	+	-	-	LS-C337258	OTI4G8	LS Bio
PKC-ε	-	-	-	-	+	+	-	-	LS-C172750	OTI3A12	LS Bio
PKC-ε	-	-	-	-	+	+	-	-	LS-C75178	22B10	LS Bio
PKC-ε	+	+	+	+	+	+	+	+	ab124806	EPR1482	Abcam
PKC-ε	+	+	+	+	+	+	+	+	Bs2329R	NA	BIOSS
PKC-ε	-	+	-	+	+	+	+	-	NSP2-03620	OTI4G8	Novus Biologicals
PKC-η	+	+	+	+	+	+	+	+	H00005583-D01P	NA	Abnova
PKC-η	+	+	+	+	+	+	+	+	H00005583-B02P	NA	Abnova
PKC-η	+	+	+	+	+	+	+	+	H00005583-D01	NA	Abnova

Cross reactivity (+), no cross reactivity (-); *Antibody utilized in western blot experiment in Figure 7.
