# Supplementary material for: Activating PKC-ε induces HIV expression with improved tolerability
Source: PLoS Pathog. 2025 Feb 6;21(2):e1012874. doi: 10.1371/journal.ppat.1012874 (PMC11801715; doi:10.1371/journal.ppat.1012874)
Supplement: S3 Table — (PDF) [file ppat.1012874.s003.pdf]

Table S3. Cytokine induction in vitro in PBMC culture supernatants treated with non-selective and selective PKC agonists

Cytokine (pg/mL)	Prostratin[a]		C-232A[a]		C-233[a]		DMSO
	625 nM	2500 nM	156 nM	625 nM	156 nM	625 nM	
GM-CSF	35	68	40	71	14	30	0.3
IFN-γ	255	292	202	172	215	135	5
IL-1b	57	108	72	125	42	60	5.5
IL-2	22	32	14	17	10	14	4
IL-4	0.7	1.1	0.4	0.6	0.3	0.6	0.1
IL-6	40	78	84	100	40	44	25
IL-8	40,698	45,930	43,224	45,964	36,739	45,465	4920
IL-10	4	4.6	4.9	3.6	4	4	1
IL-12p70	10	12	7	9	5	9	2
TNF-α	634	1489	883	1424	308	834	4

[a]Cytokine levels induced at concentrations equivalent to 0.7x and 3x EC_{50} (Prostratin), 1.7× and 7× EC_{50} (C-232A) or 1.2× and 5× EC_{50} (C-233) values for HIV reactivation (Figure 4 and 10) are shown.
