# Supplementary figures and images for: Activating PKC-ε induces HIV expression with improved tolerability
Source: PLoS Pathog. 2025 Feb 6;21(2):e1012874. doi: 10.1371/journal.ppat.1012874 (PMC11801715; doi:10.1371/journal.ppat.1012874)

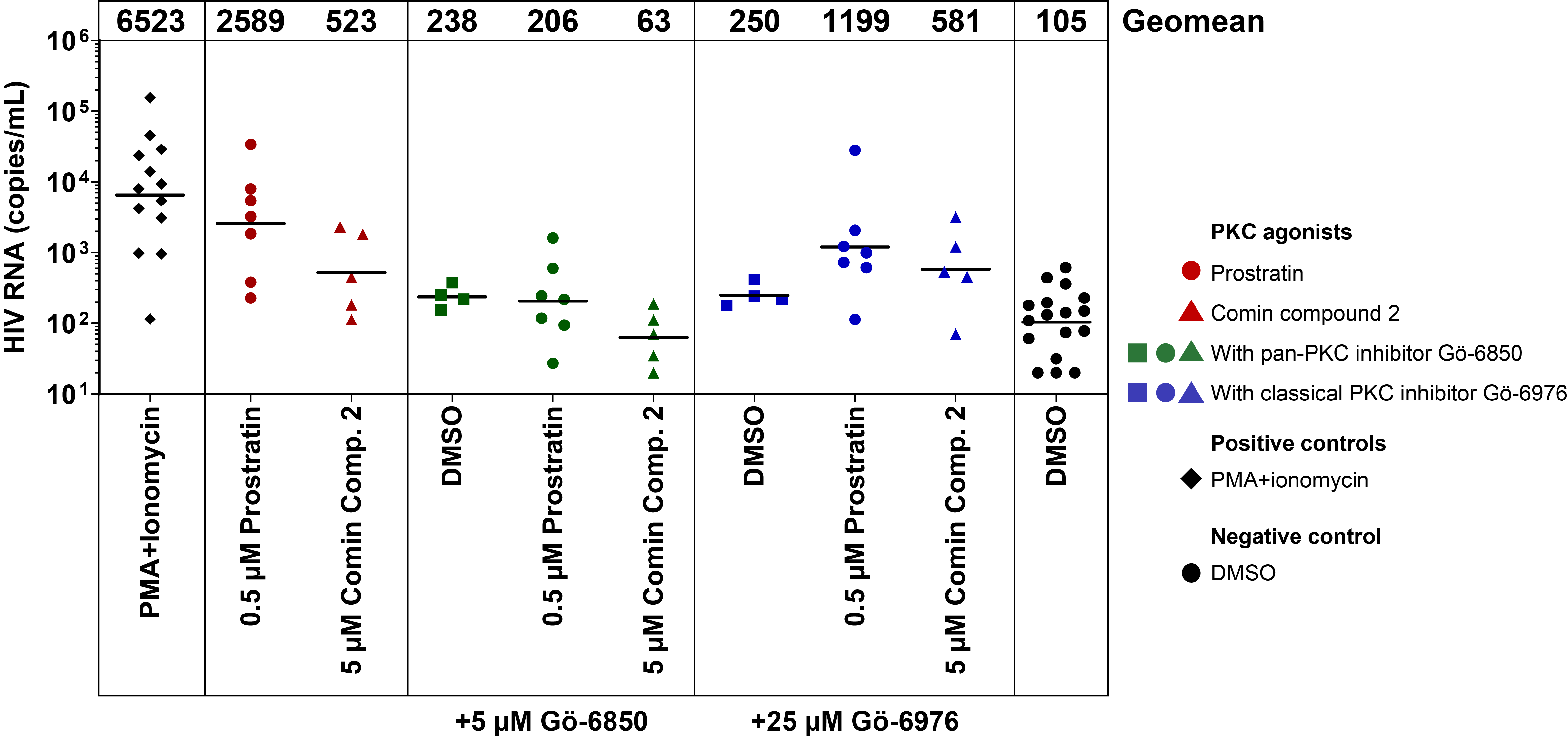

Supplement: S1 Fig — Figure representing untransformed HIV RNA values from Fig 2. HIV viral RNA (copies/mL) in total CD4+ T cells from ART-suppressed people with HIV (n = 12) treated with PKC agonists (prostratin [red circles, n = 7]/Comin compound 2 [red triangles, n = 5]) alone, with pan-PKC inhibitor Gӧ-6850 (green circles/green triangles), or with classical PKC inhibitor Gӧ-6976 (blue circles/blue triangles). PMA and ionomycin (black diamonds) were used as positive control. DMSO (vehicle) was used as negative control (black circles and green/blue squares). Each symbol represents the geometric mean for a single individual (n = 3 to 8 replicate wells). Horizontal bars indicate the geometric means of all individuals in the indicated condition. (PNG) [file ppat.1012874.s004.png]

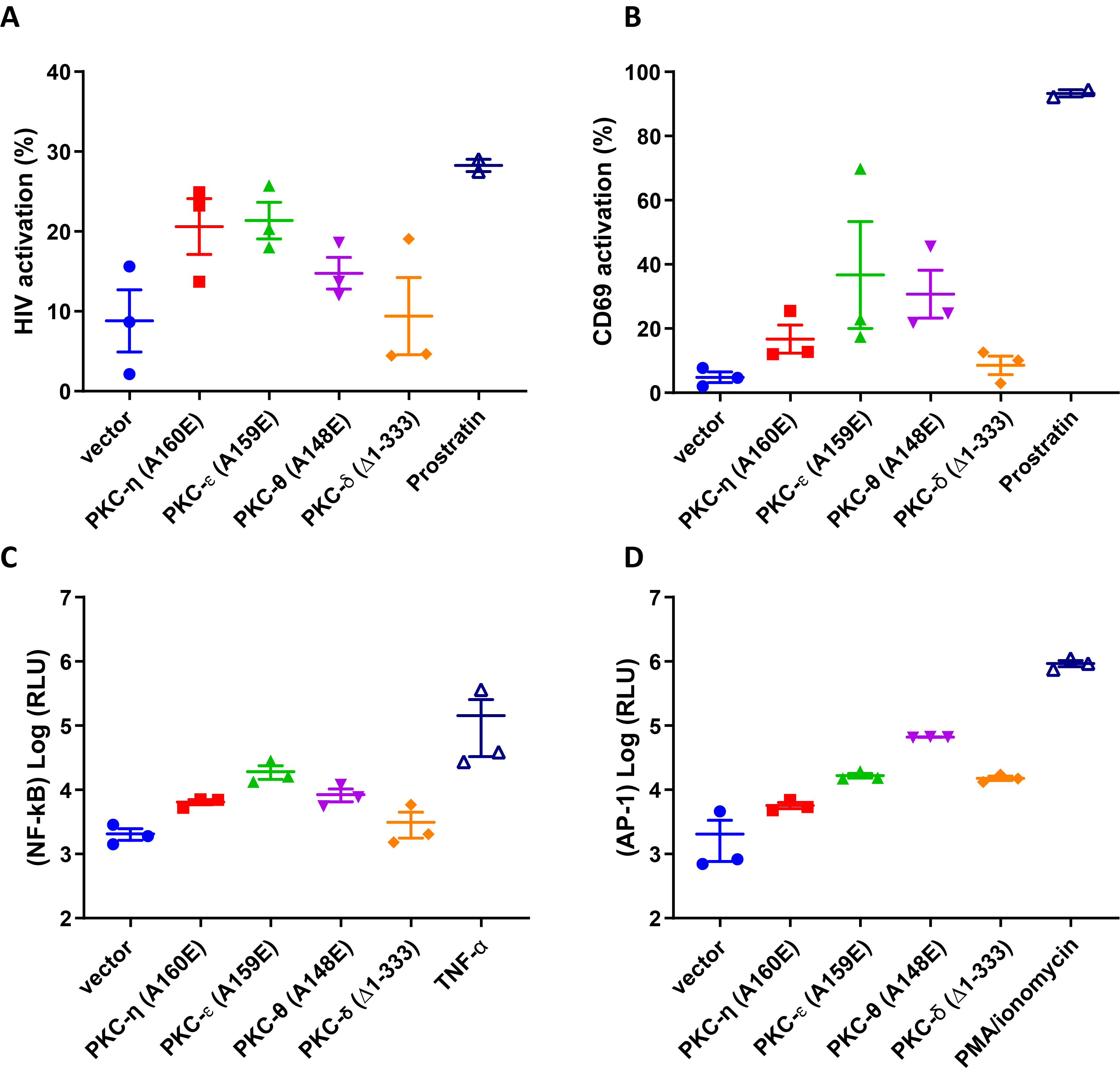

Supplement: S2 Fig — (A–B) HIV and CD69 activation in latent Jurkat latent reporter cell line expressing constitutively active PKC-ε, η, θ, and δ. Percent HIV expression was measured in Jurkat cell line latently infected with HIV-1 NL4-3 Gag-iGFP Δenv and transiently transfected with constitutively active novel PKC isoforms ε, η, θ or δ utilizing pcDNA3.1. Expression was measured at 48 hours post transfection by gating on GFP+ (positive for HIV activation) and RFP+ (positive for transfected and PKC isoform expressing) cells. Prostratin was a positive control representing cells treated with 0.4 μM prostratin for 48 hours alone without any transfection. (A) The percent HIV activation obtained under each condition. (B) Percent CD69 expression at 48 hours in the same cells indicating activation upon expression of constitutively active novel PKC isoforms. Expression of luciferase driven by promoters responsive to NF-kB (C) and AP-1 (D) in HEK293 cells expressing constitutively active novel PKC isoforms. Transfected cells were incubated for 48 hours, and luciferase activity was measured using One-Step Luciferase Assay. (PNG) [file ppat.1012874.s005.png]

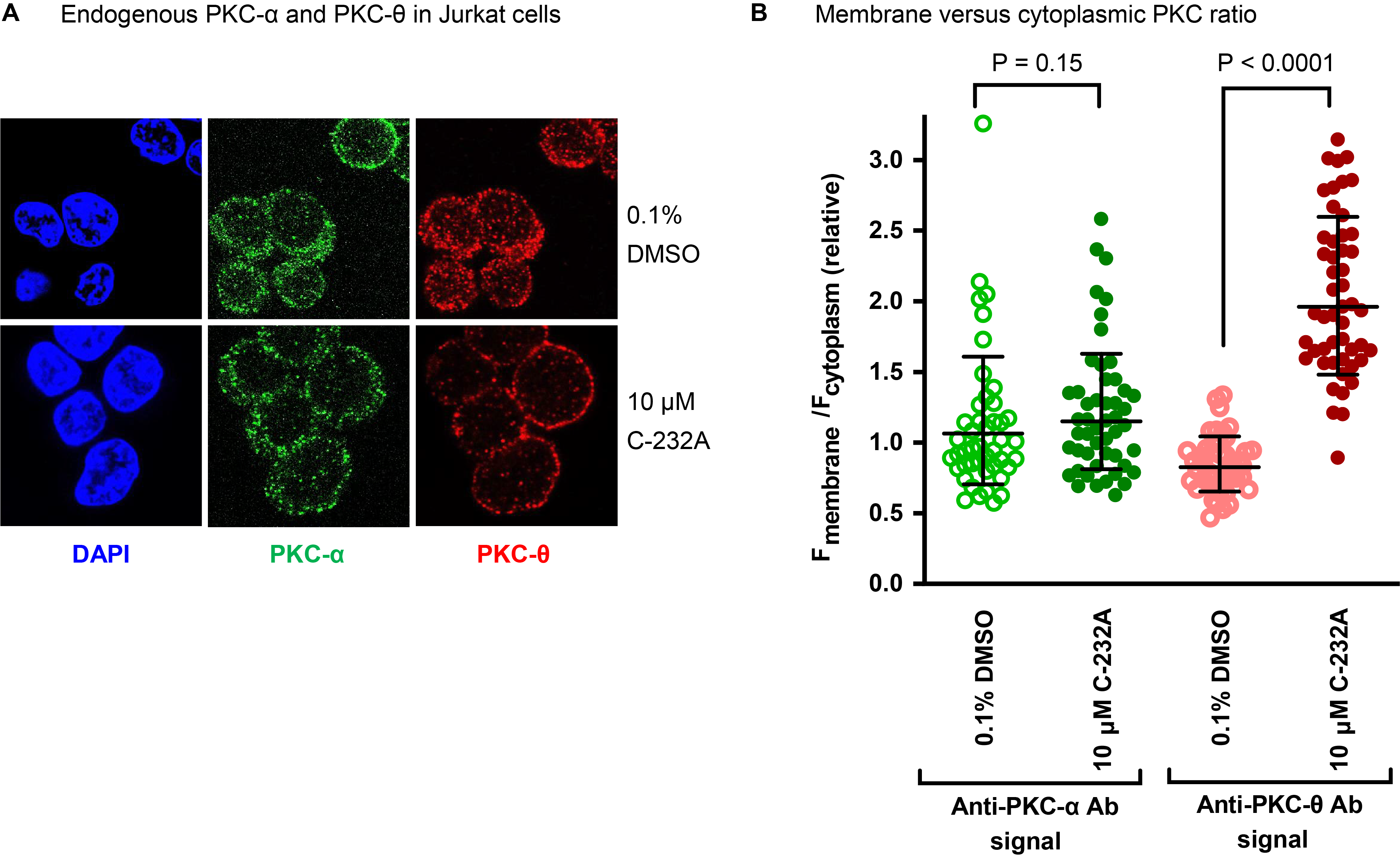

Supplement: S3 Fig — (A) Confocal images of immunostained Jurkat cells showing translocation of endogenous PKC-θ (bottom panel/right) from cytoplasm to cell membrane upon 30-minute treatment with C-232A but not PKC-α (bottom panel, middle). Clear translocation of PKC-θ from cytoplasm to cell edge can be seen despite the small distance between plasma membrane and nuclear envelope in C-232A treated samples. Cells treated with vehicle control, DMSO, do not show translocation of either of these isoforms (top, right and middle). DAPI staining indicates (left panels) nuclear location within each cell. (B) Translocation efficiency of PKC isoforms was quantified using the ratio between the average fluorescence at the cell edge versus the cytoplasmic region. Significant increase in this ratio is seen for PKC-θ in cells treated with C-232A (red fluorescence) compared with PKC-α (green fluorescence), suggesting that C-232A effectively activated and translocated novel isoform PKC-θ but not PKC-α. Cells treated with vehicle control, DMSO, do not show translocation of either of these isoforms. Each individual data point represents the ratio of fluorescent signal from individual cells that were analyzed. Horizontal bars represent geometrical means with standard deviation and P values were obtained using Mann–Whitney test. (PNG) [file ppat.1012874.s006.png]

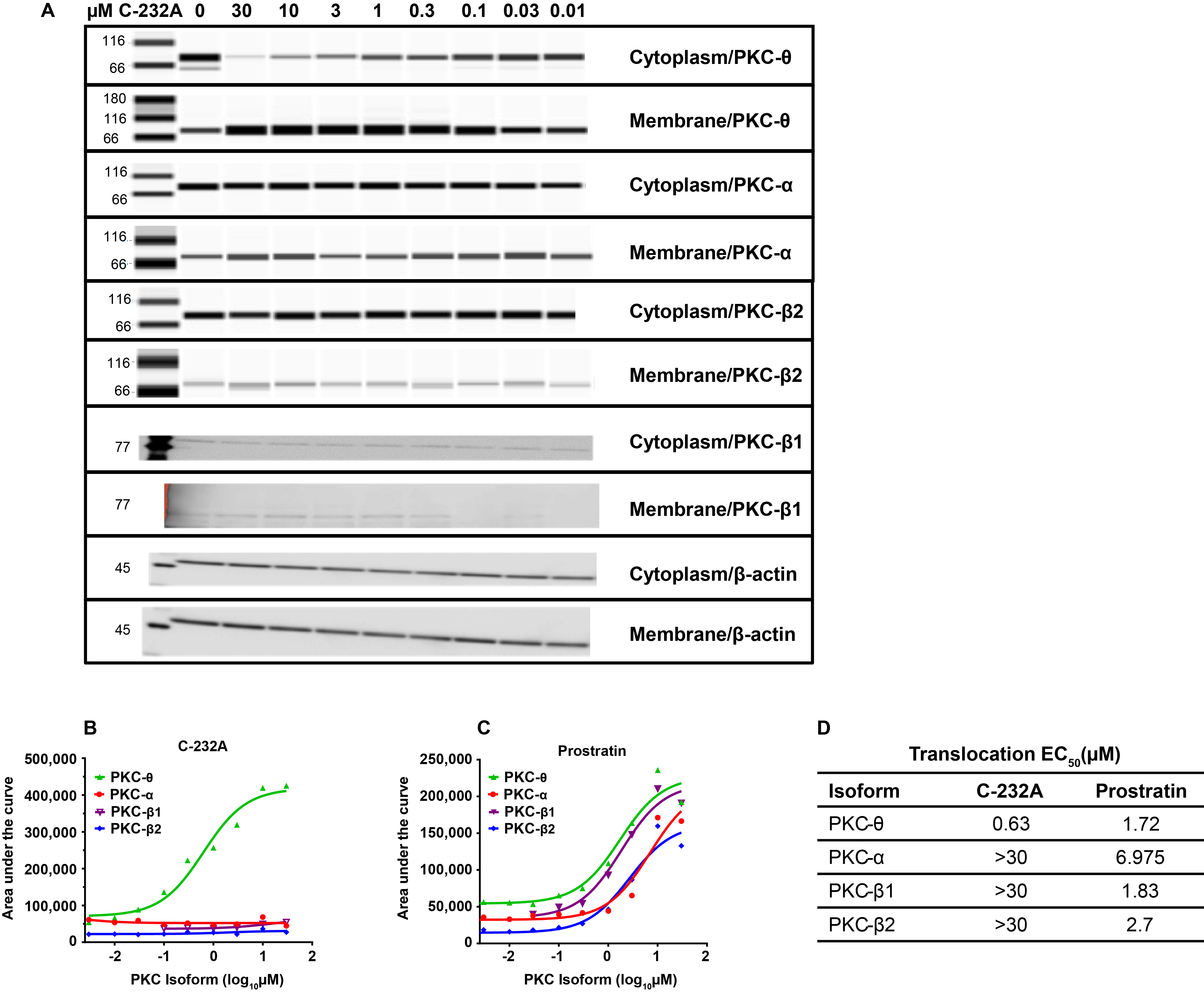

Supplement: S4 Fig — (A) Western blot of PKC isoforms θ, α, β2, and β1 in cytoplasmic and membrane fractions of CD4+ T cells treated with C-232A. Quantitative measurement of translocation of PKC isoforms by C-232A (B) and prostratin (C) in CD4+ T cells. Area under the peak generated by western blot analysis of membrane-associated PKC isoforms after treatment and plotted using GraphPad software. AUC plots were used to generate EC50 values (D). (PNG) [file ppat.1012874.s007.png]

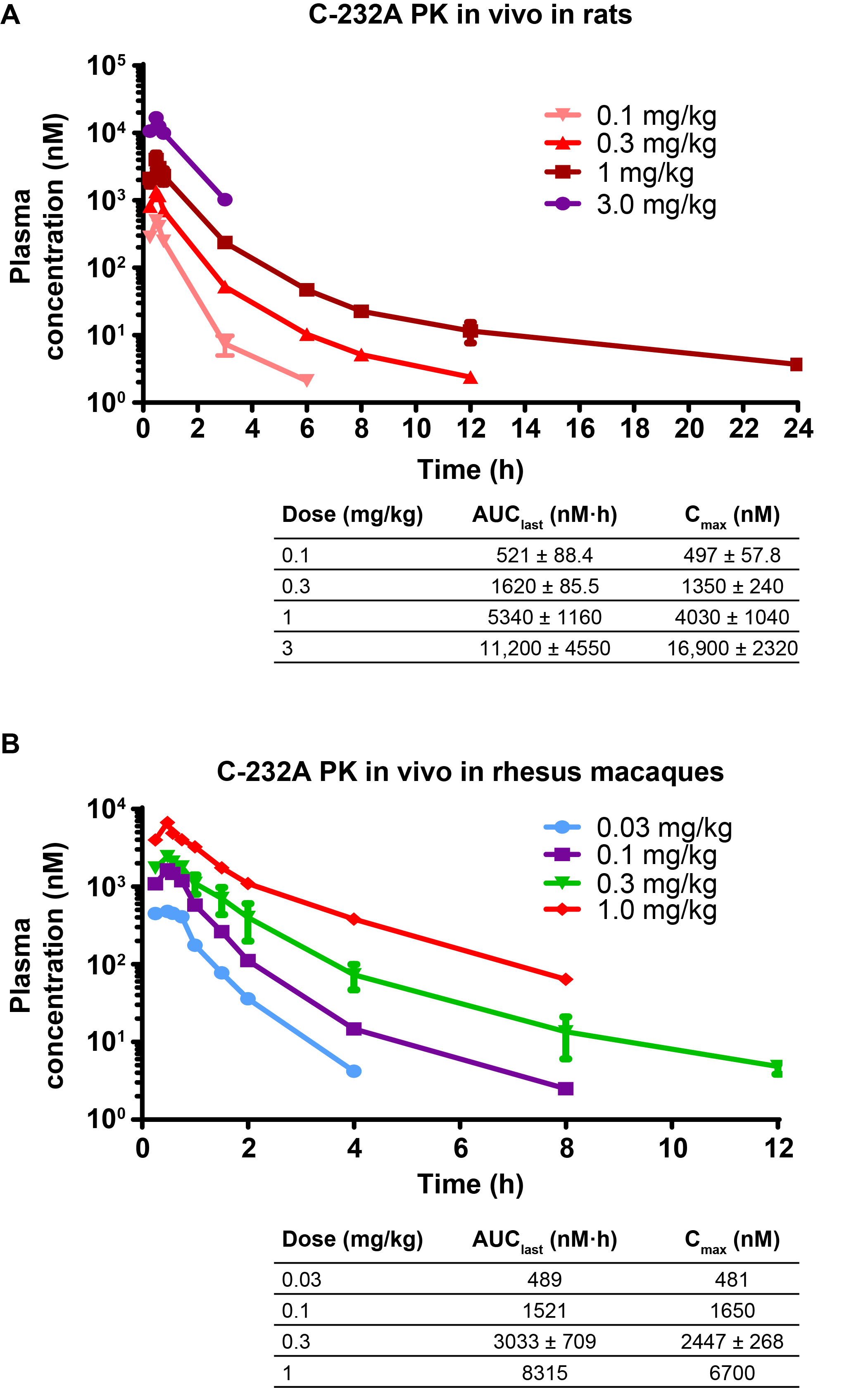

Supplement: S5 Fig — (A) Plasma concentrations of C-232A from dose escalation studies in Sprague Dawley rats, dosed by slow IV infusion. Rats were dosed at 0.1, 0.3, 1, and 3 mg/kg and plasma concentrations of C-232A were measured over a time period of 24 hours after dosing. (B) Plasma concentrations of C-232A from dose escalation studies in rhesus macaques, dosed by slow, IV infusion. Rhesus were dosed at 0.03, 0.1, 0.3, and 1 mg/kg and plasma concentrations of C-232A were measured over a time period of 24 hours after dosing. (PNG) [file ppat.1012874.s008.png]

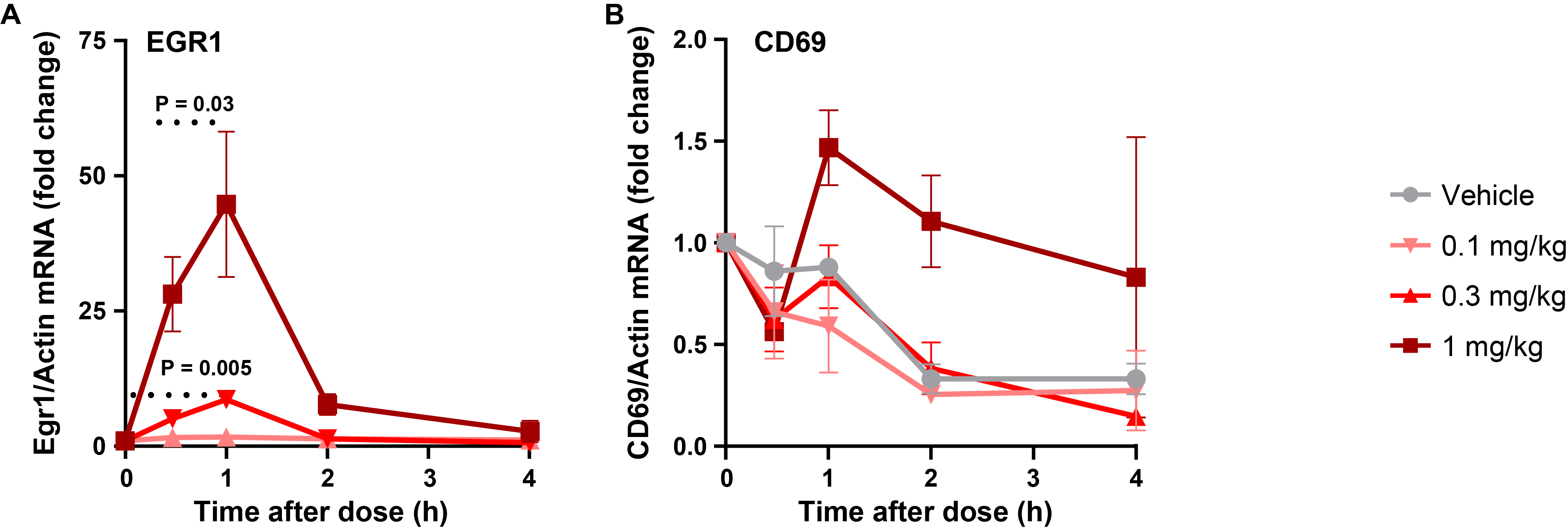

Supplement: S6 Fig — Dose-dependent expression of EGR1 (A) and CD69 (B) mRNA levels measured by QuantiGene analysis in rat whole blood (n = 3) at different time points after C-232A dosing. The y-axis indicates mean fold change in post-dose expression levels compared with pre-dose after normalizing to β-actin mRNA levels. Mean values are plotted with error bars indicating standard deviation. P values were calculated using paired t test. (PNG) [file ppat.1012874.s009.png]

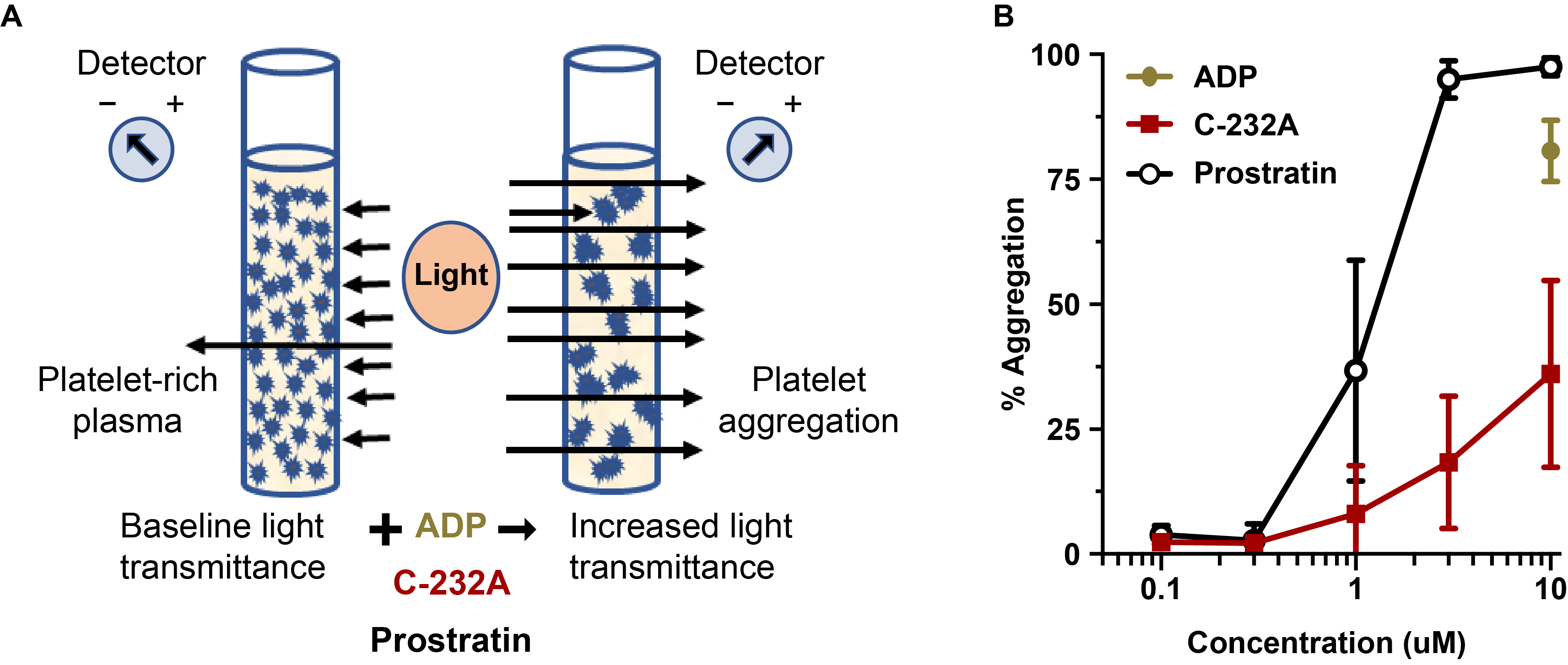

Supplement: S7 Fig — (A) Assay principle for platelet aggregation assay. Platelet rich plasma made from fresh whole blood from healthy donors (n = 3) was treated with either C-232A or prostratin in a dilution series for 20 minutes and rate of light transmission before and after treatment was measured by light transmittance aggregometry. (B) In vitro platelet aggregation caused by prostratin (black open circles), C-232A (red squares), and an assay positive control (adenosine diphosphate [ADP]; (brown circles). (PNG) [file ppat.1012874.s010.png]

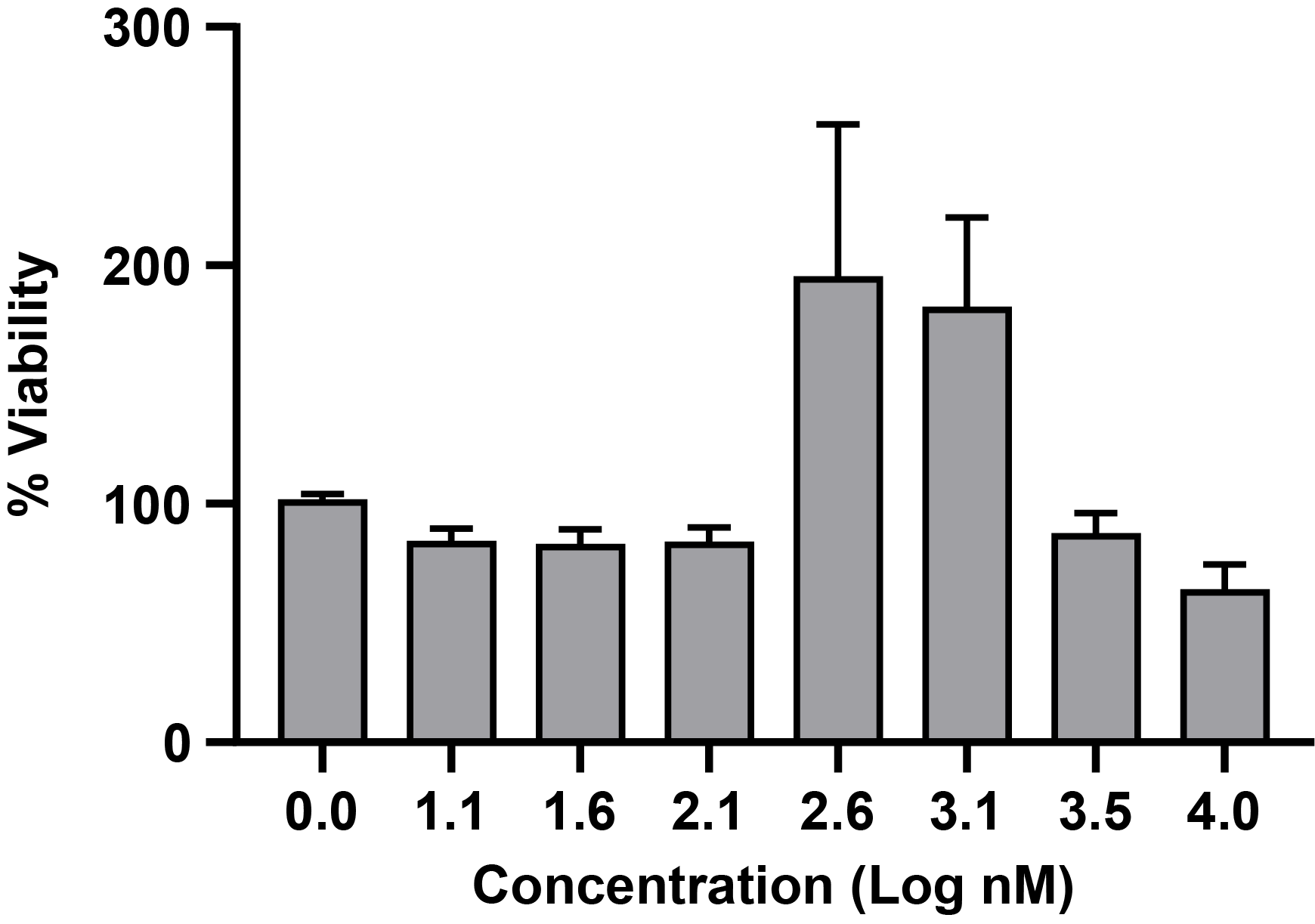

Supplement: S8 Fig — CD4+ T cells from ART-suppressed people with HIV were treated with the indicated C-233 concentrations for 72 hours. Cell viability was assessed using Promega Cell Titer Glo according to manufacturer’s instructions. Bars indicate the means and lines indicate standard deviation from 4 donors, each tested with 8 replicates. (PNG) [file ppat.1012874.s011.png]

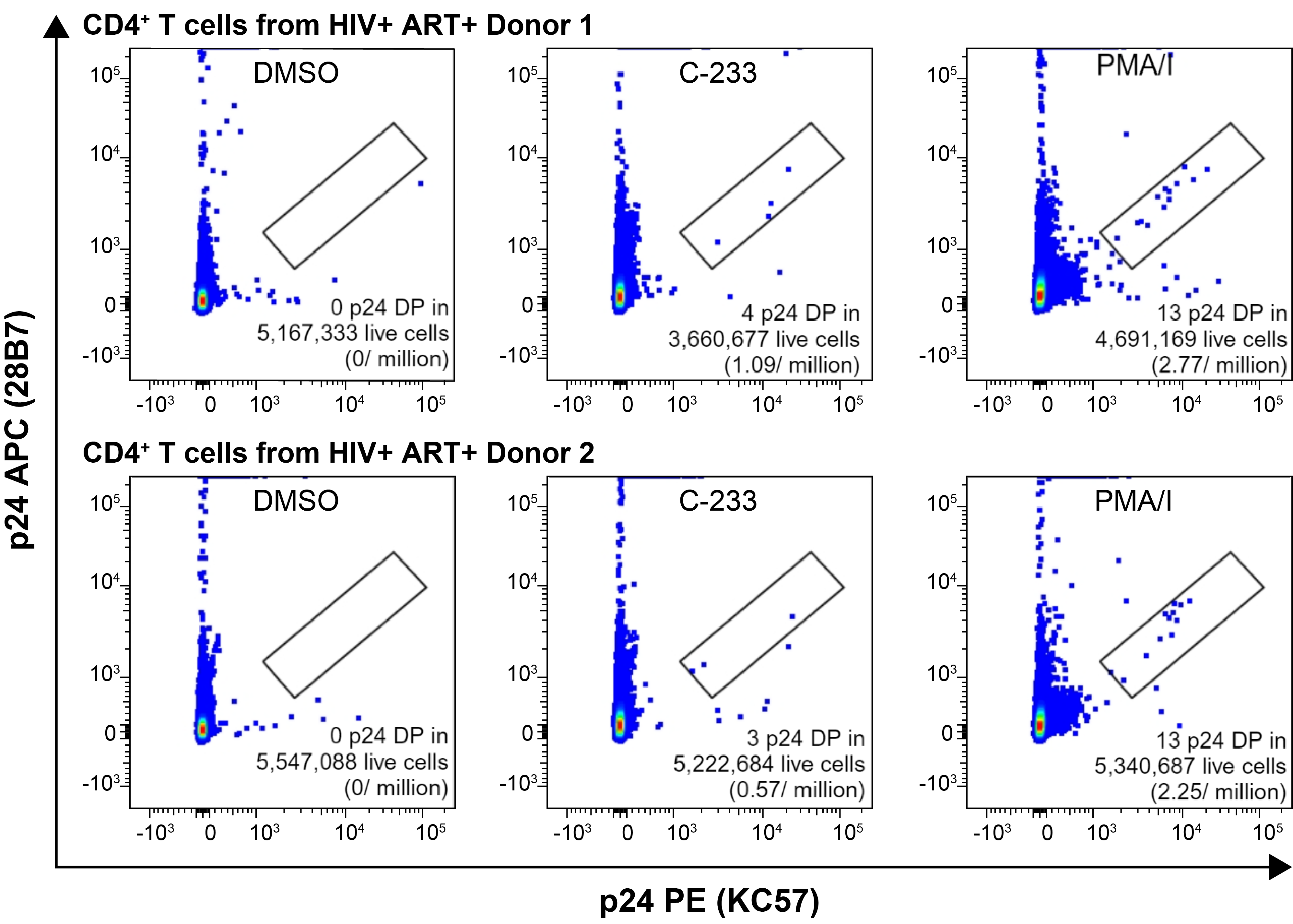

Supplement: S9 Fig — Dot plots showing HIV-Flow anti-p24-PE (KC57) and anti-p24-APC (28B7) antibody co-staining in CD4+ T cells isolated from 2 ART-suppressed individuals with HIV treated with DMSO (control), 500 nM C-233, or 162 nM PMA and 1 µg/mL ionomycin for 72 hours. The indicated gates define the p24 double positive (p24 DP) population of reactivated HIV-infected cells. (PNG) [file ppat.1012874.s012.png]

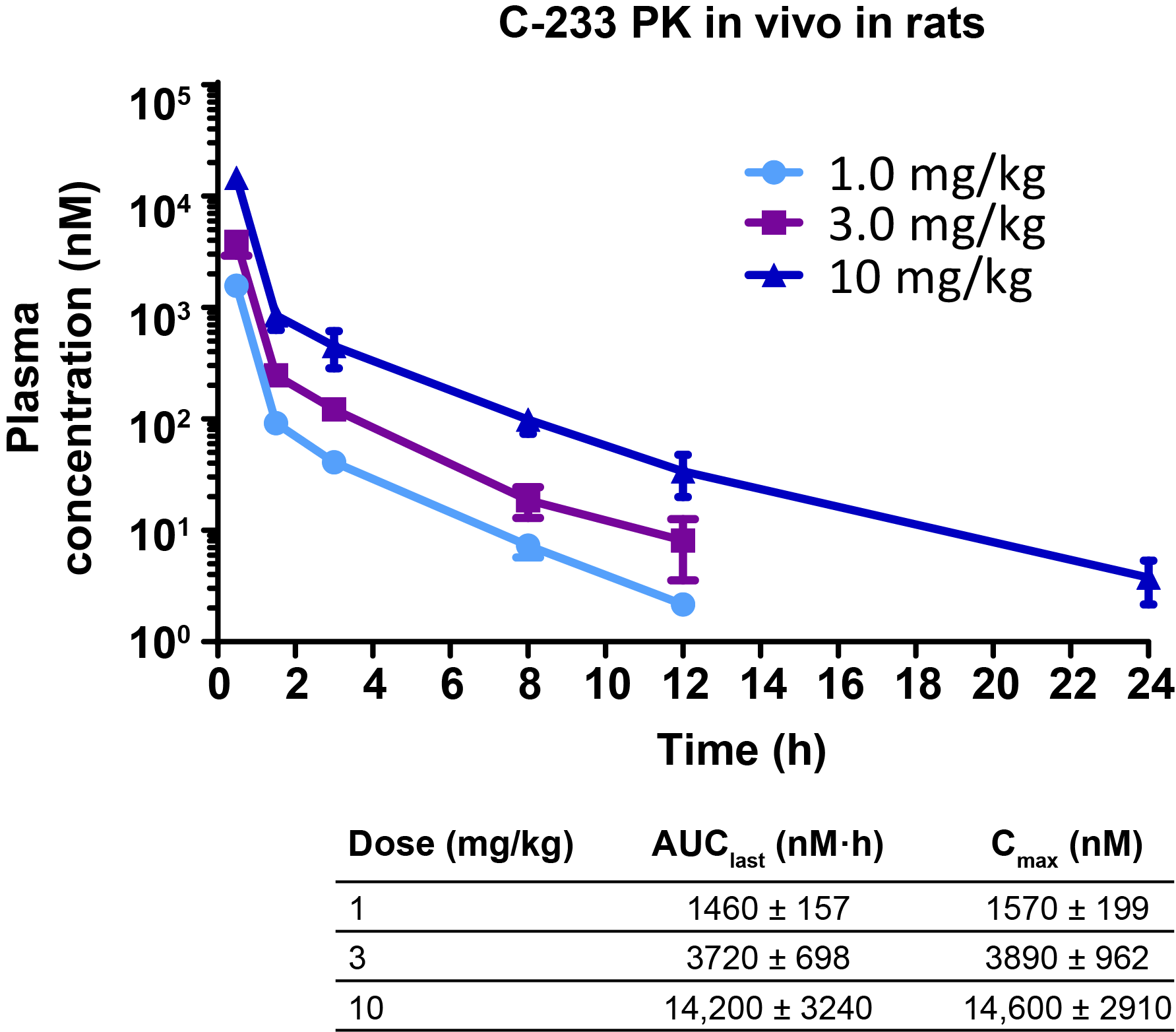

Supplement: S10 Fig — Plasma concentrations of C-233 from dose escalation studies in Sprague Dawley rats, dosed by slow IV infusion. Rats (n = 3) were dosed at 1, 3, and 10 mg/kg and plasma concentrations of C-233 were measured for 24 hours after dosing. After in vivo administration, dose-dependent concentrations of C-233 in plasma were noted. (PNG) [file ppat.1012874.s013.png]

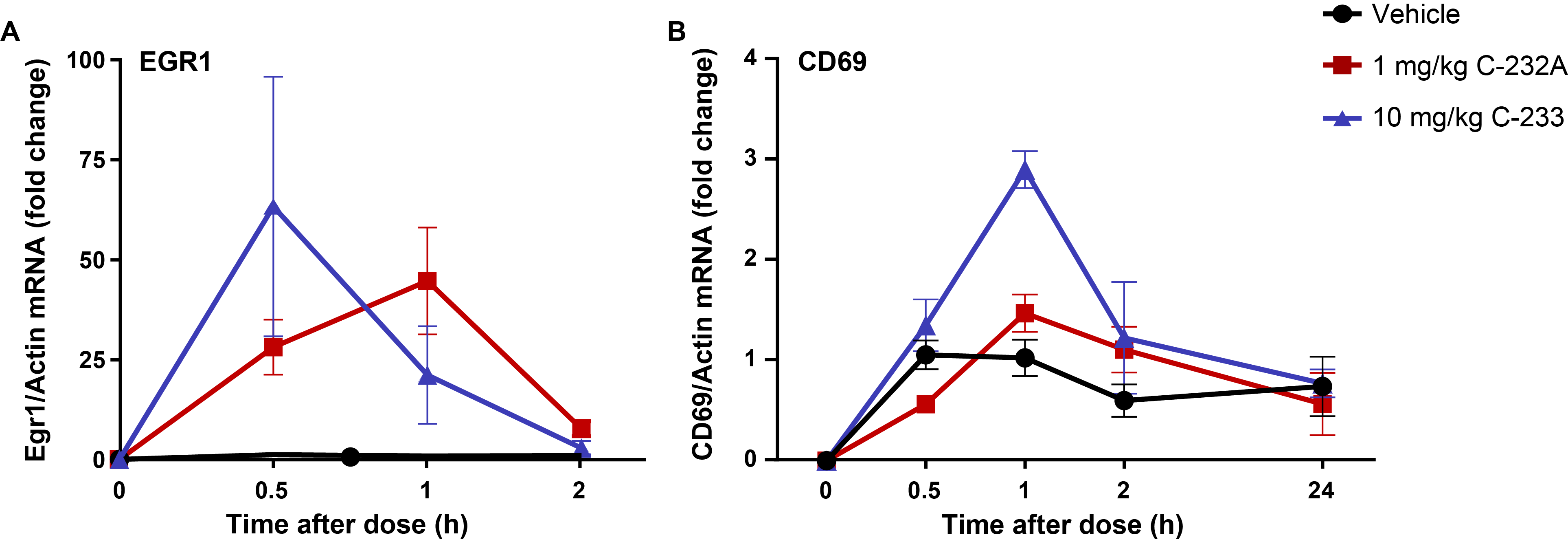

Supplement: S11 Fig — Expression levels of (A) Egr1 and (B) CD69 mRNA levels measured by QuantiGene analysis in rat whole blood at different time points after C-233 (10 mg/kg) dosing in comparison with C-232A (1 mg/kg). Expression levels were normalized to predose values after normalizing to β-actin mRNA levels (n = 3). (PNG) [file ppat.1012874.s014.png]
